# Tubular Bridges for Bronchial Epithelial Cell Migration and Communication

**DOI:** 10.1371/journal.pone.0008930

**Published:** 2010-01-28

**Authors:** Brett G. Zani, Laura Indolfi, Elazer R. Edelman

**Affiliations:** 1 Harvard-MIT Division of Health Sciences and Technology, Massachusetts Institute of Technology, Cambridge, Massachusetts, United States of America; 2 Interdisciplinary Research Center on Biomaterials (CRIB) - Italian Institute of Technology, University of Naples Federico II, Naples, Italy; 3 Cardiovascular Division, Brigham and Women's Hospital, Harvard Medical School, Boston, Massachusetts, United States of America; University of Giessen Lung Center, Germany

## Abstract

**Background:**

Biological processes from embryogenesis to tumorigenesis rely on the coordinated coalescence of cells and synchronized cell-to-cell communication. Intercellular signaling enables cell masses to communicate through endocrine pathways at a distance or by direct contact over shorter dimensions. Cellular bridges, the longest direct connections between cells, facilitate transfer of cellular signals and components over hundreds of microns in vitro and in vivo.

**Methodology/Principal Findings:**

Using various cellular imaging techniques on human tissue cultures, we identified two types of tubular, bronchial epithelial (EP) connections, up to a millimeter in length, designated EP bridges. Structurally distinct from other cellular connections, the first type of EP bridge may mediate transport of cellular material between cells, while the second type of EP bridge is functionally distinct from all other cellular connections by mediating migration of epithelial cells between EP masses. Morphological and biochemical interactions with other cell types differentially regulated the nuclear factor-κB and cyclooxygenase inflammatory pathways, resulting in increased levels of inflammatory molecules that impeded EP bridge formation. Pharmacologic inhibition of these inflammatory pathways caused increased morphological and mobility changes stimulating the biogenesis of EP bridges, in part through the upregulation of reactive oxygen species pathways.

**Conclusions/Significance:**

EP bridge formation appears to be a normal response of EP physiology in vitro, which is differentially inhibited by inflammatory cellular pathways depending upon the morphological and biochemical interactions between EP cells and other cell types. These tubular EP conduits may represent an ultra long-range form of direct intercellular communication and a completely new mechanism of tissue-mediated cell migration.

## Introduction

Diverse modes of intercellular communication have evolved to coordinate the physiology of multi-cellular organisms. Varied communication mechanisms via physical intercellular connectivity have been elucidated, including gap junctions and intercellular bridges [1], [2]. In mammals and invertebrates, intercellular bridges transiently connect cells preceding abscission at the termination of cytokinesis. While stable intercellular bridges form at the end of germ cell cytokinesis creating an interconnected syncytium of daughter cells [3]. Recently characterized cellular bridges - cytonemes and tunneling nanotubes (TNTs) - facilitate transfer of cellular signals and components, even pathogens, over hundreds of microns representing the longest direct connections between cells in vitro and in vivo [4]–[9].

Found in vertebrate and invertebrate cells, cytonemes are not tubes but connect neighboring cells allowing for signal transduction and transport of cellular molecules along the outer structural surface. These F-actin-based structures have a maximum width of 200 nm and measure up to 700 µm in length, with most cytonemes being shorter than 100 µm [4], [10], [11]. In contrast to cytonemes, F-actin-rich TNTs are membranous tubular conduits facilitating direct intercellular transfer of organelles, cytoplasmic molecules, and membrane components. Pathogens, such as prions and retroviruses, also use TNTs to promote spreading between cells [6], [8], [9]. TNTs, which are 50–200 nm in diameter and tens to hundreds of µm in length, hover above while making no contact with the substratum, and are dynamic – usually remaining intact for only minutes to several hours [5], [12].

In tissue cultures of primary human cells - bronchial epithelial cells (EPs) cultured with aortic endothelial cells (ECs) or lung fibroblasts (FBs) – we observed two types of tubular, bronchial epithelial (EP) connections between EPs. Designated EP bridges, these cellular connections were structurally distinct from cytonemes and TNTs, and could possess far greater length and permanence. The first type of EP bridge may be functionally similar to cytonemes and TNTs by possibly mediating transport of cellular material between cells, while the second type of EP bridge is distinct in function compared to cytonemes and TNTs by facilitating the migration of entire cells between EP masses. EP bridge formation was impeded by increased levels of inflammatory molecules resulting from EP morphological and biochemical interactions with ECs or FBs that differentially regulated the nuclear factor (NF)-κB and cyclooxygenase (COX) inflammatory pathways. Formation of EP bridges was increased by blocking these inflammatory pathways, in part through upregulation of reactive oxygen species (ROS). Based on these findings, EP bridges appear to be a normal aspect of EP physiology in vitro that can be diminished by inflammation. The structure, function, and regulation of EP bridges present novel forms of cellular connections that may facilitate direct intercellular communication over the longest distances reported to date and mediate an entirely new mechanism of tissue-mediated cell migration.

## Results and Discussion

**Figure 1:**
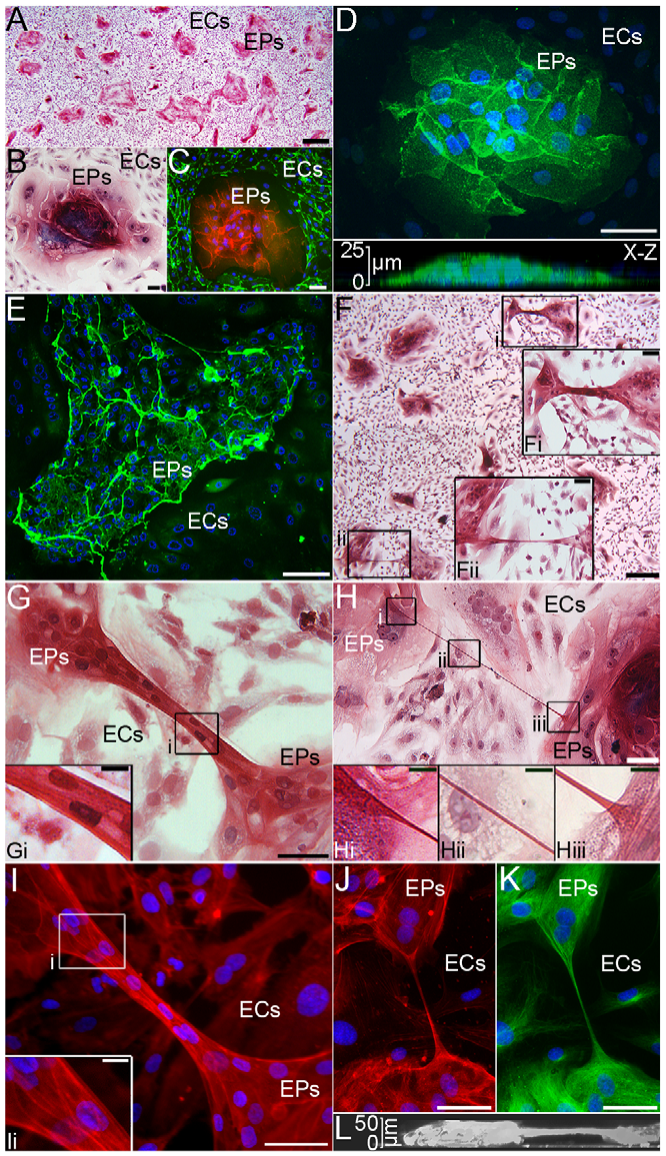
Segregation and EP bridge architecture in EPs/ECs. **A–B:** Trichome staining showed EP islands surrounded by ECs. **C:** CD31 (green), E-cadherin (red), and nucleus (blue) immunostaining of distinct boundaries between cell types. **D:** Confocal imaging of an EP island immunostained for E-cadherin (green) and nuclei (blue) with 3D reconstruction shown in an x-z section. **E:** EP island barriers were composed of collagen IV (green) **F:** EP bridges with (Fi) or without nuclei (Fii) connected EP islands. **G–H:** Trichrome staining of EP bridges with nuclei (E) and without nuclei (F). **I–K:** F-actin (red), nucleus (blue), and acetylated α-tubulin (green) immunostaining for microtubules showed EP bridge structural components. **L:** x-z section of an EP bridge from confocal 3D reconstruction. Scale bars: (A), 500 µm; (B–E, Fi–Fii), 50 µm; (F), 250 µm; (G–K), 50 µm; (Gi, Hi–Hiii, Ii), 10 µm.

As mixed co-cultures of EPs and ECs approached confluence on tissue culture plates, an organized array formed with monolayers of ECs surrounding complex, multi-layered EP masses referred to here as EP islands (Figure 1, A to C). Cysts, somewhat similar in appearance to alveolar sacs in vivo, formed within EP islands (Figure S1, A to D). Cell specific markers for differentiated basal (Cytokeratin 5) and secretory (CC10, MUC5AC) EPs revealed that non-mucus, secretory Clara cells partially compose EP islands with the remaining composition of EP islands appearing to be undifferentiated, transitional EPs (Figure S1, E and F). Confocal microscopy showed EP islands could reach at least 25 to 50 µm in height as EPs grew one on top of another (Figure 1D). The natural segregation of human adult cell types and the emergence of multi-cellular formations in co-cultures mimicked studies where fetal mouse lungs were dissociated and cultured [13], [14]. In these prior studies, fetal mouse EPs, ECs, and mesenchymal cells recombined, sorted, polarized, branched, and secreted extracellular matrix to form EP cysts surrounded by mesenchyme with endothelial networks branching throughout the multi-cellular system. Morphological analysis in our co-cultures revealed distinct cellular barriers composed of collagen IV between EP islands and ECs (Figure 1E). The intact airway architecture in vivo includes a layer of connective tissue that constitutes the lamina propria which forms a physical barrier between the epithelium and the underlying mesenchyme [15]. The formation of distinct connective tissue barriers between EP islands and ECs may be an attempt by EPs to form a lamina propria in vitro.

**Figure 2:**
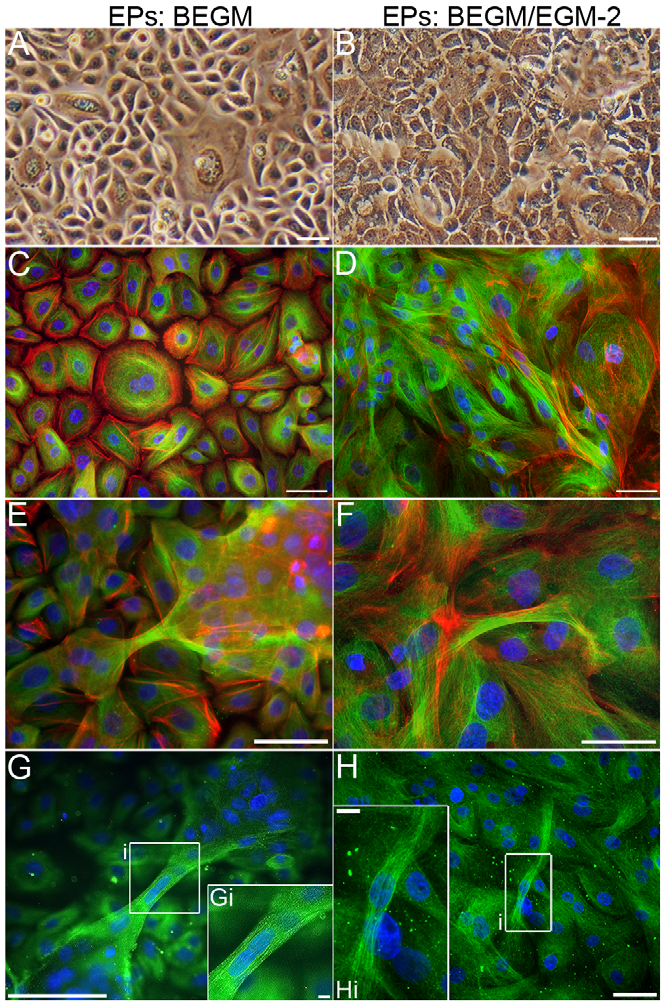
Medium affects morphology of EPs and EP bridges. **A–B:** Photomicrographs of EPs cultured alone in BEGM (A) depict a cuboidal EP morphology compared to a flattened, squamous morphology displayed in EPs grown in BEGM:EGM-2 with 5% FBS (B). **C–D:** Composites of microtubules via acetylated α-tubulin (green), F-actin (red), and nucleus (blue) immunostaining in EPs cultured alone in BEGM (C) show a more organized cytoskeleton compared to EPs grown in BEGM:EGM-2 with 5% FBS (D). **E–H:** Expression of microtubules (green) and F-actin (red) in EP bridges without nuclei (E–F) or with a nucleus (G–H) in EPs cultured alone in BEGM (E, G) or BEGM:EGM-2 with 5% FBS (F, H). Scale bars: (A–G), 50 µm; (Gi, Hi), 10 µm.

Long, thin EP structures - EP bridges - established connections between EP islands (Figure 1, F to H). Some EP bridges contained nuclei, while all EP bridges were F-actin and microtubule composites (Figure 1, I to K), in contrast to cytonemes and TNTs which contain only F-actin [4], [5]. EP bridge formation was not dependent on the confluence of co-cultures as these structures were observed forming both before and after confluence. Similar to TNT structure, EP bridges hovered above the underlying substratum of ECs (Figure 1L). Possessing both dynamic and static morphological characteristics, EP bridges remained in place up to 2 days and ranged from 50 µm to over a millimeter in length. Time-lapse light microscopy showed EP bridges extending (Figure S2) or contracting (Movie S1) as EP islands moved apart or together. EP bridges were also much wider than cytonemes or TNTs with diameters of 1 to 20 µm. Furthermore, EP bridge formation was not predicated on the presence of ECs as EP bridges were observed in EPs cultured with FBs (Figure S3) or EPs cultured alone (Figure 2).

The elongated shapes of EP bridges raised the possibility EPs were undergoing epithelial-to-mesenchymal transition (EMT). During development, tumorigenesis, and fibrosis in various tissues, cellular transdifferentiation of EPs to fully differentiated FBs or myofibroblasts can occur via EMT [16], [17]. EPs transition morphologically from cuboidal shapes to spindle or elongated shapes, lose epithelial-specific markers such as E-cadherin, and express mesenchymal-specific markers such as α-smooth muscle actin and vimentin. However, EPs and EP bridges in all culture conditions strongly expressed E-cadherin and did not express α-smooth muscle actin (Figure S4) or vimentin (Figure S5); implying EP bridges are epithelial in nature.

For direct comparisons among co-cultures and EPs cultured alone, all cultures were grown in the same medium - a 1∶1 mixture of bronchial epithelial growth medium (BEGM) and endothelial growth medium-2 (EGM-2) with a total of 5% fetal bovine serum (FBS). FBS can induce terminal squamous differentiation in EPs [18]. Indeed, EPs exposed to 5% FBS in BEGM:EGM-2 displayed a flattened, squamous morphology with a less organized F-actin and microtubule cytoskeleton compared to EPs that displayed a cuboidal morphology when not exposed to FBS in BEGM alone (Figure 2). Even with these differences in EP morphology, EP bridges formed with or without exposure to FBS. Although 48 hours after cultures became confluent in a 4 cm^2^ culture well, significantly more EP bridges (117.3±18.2) formed in EPs grown in BEGM alone than in EPs exposed to FBS in the mixed growth medium (56.0±2.6, Table 1), suggesting FBS does affect EP bridge formation. Yet, the existence of EP bridges in both growth mediums suggests EP bridge formation is a natural process of EP physiology, at least in vitro. In support of this notion, preliminary studies in a more physiologically relevant model showed EP bridge formation with EPs exposed to air on the apical surface of an air-liquid interface with FBs grown on the basal surface (Figure S6). Existence in vivo will be the ultimate determinant of any physiological relevance for EP bridges; however, crucial insights can be gleaned from in vitro studies characterizing EP bridge morphology and function and the regulation of EP bridge formation from interactions with other cell types and soluble factors.

**Table 1:** EP bridge characterization in 48 h post-confluent cultures.

Culture Type	Bridges/Well^1^	Bridge Length, µm	Length Range, µm	Type II Bridges (%)^2^
EPs	117.3±18.2*	93.7±3.0	26–293	18.8*
(BEGM); n =	3	233		
EPs	56.0±2.6#	103.6±3.3	36–313	1.8
(Mix); n =	3	168		
EPs/ECs	19.9±2.6	193.1±7.6†	51–690	40.6†
(Mix); n =	20	196		
EPs/FBs	57.0±5.4#	179.5±4.9†	27–633	44.0†
(Mix); n =	19	384		

1 Average number of total EP bridges per 4 cm^2^ tissue culture well.

2 Percentage of Type II EP bridges based on the total number of EP bridges.

*p<0.05 vs. EPs (Mix), EPs/ECs, and EPs/FBs; #p<0.05 vs. EPs (BEGM) and EPs/ECs; †p<0.05 vs. EPs (BEGM) and EPs (Mix).

Mix  =  cells were grown in mixed culture medium of BEGM:EGM-2 with 5% FBS, while BEGM has no FBS.

In fact, EP bridges in EPs/ECs displayed different characteristics than in EPs/FBs, suggesting morphological and biochemical interactions of EPs with other cell types differentially regulate EP bridge formation. In co-cultures 48 hours post-confluence, 57.0±5.4 EP bridges formed in EPs/FBs, while EP bridge formation in EPs/ECs was still evident but at a significantly lower density of 19.9±2.6 EP bridges (Table 1). EP bridges in EPs cultured alone were significantly shorter than those found when EPs were co-cultured with another cell type. The longest EP bridge measured was 801 µm in EPs/ECs, but only 313 µm in cultures of EPs after fixation. EP bridges longer than a millimeter were measured during time-lapse light microscopy in co-cultures (Figure S2), but may have been too fragile at these longer lengths to survive fixation. The longer lengths of EP bridges in co-cultures versus EPs cultured alone may in part be due to the greater distances EP bridges must extend in connecting with other EPs. Also, fewer EP bridges (1.8% with FBS and 18.8% without FBS) in EPs cultured alone contained nuclei, while 40.6% to 44.0% of bridges were nucleated in co-cultures. These disparate characteristics suggest EP bridges may serve multiple potential purposes and that form and function follow local needs as defined and limited by morphological and biochemical interactions with other local cells.

**Figure 3:**
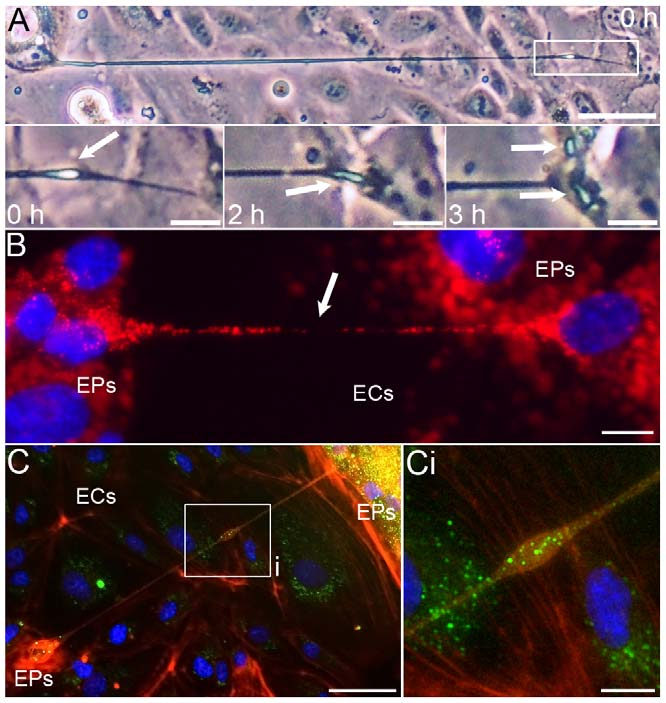
Possible transfer of cellular components via EP bridges. **A:** Time lapse light microscopy in EPs/ECs of a bleb-like structure, possibly a vesicle, moving through an EP bridge and splitting once within the EP island, arrows indicate vesicle position over 3 h. **B:** Lysosome (red) and nucleus (blue) staining in EPs/ECs of an EP bridge connecting two cells. **C:** Vesicle-like structure (Ci) within an EP bridge in EPs/ECs immunostained for F-actin (red) and a Golgi apparatus marker (green), but did not stain for the presence of DNA (blue). Scale bars: (A 0 h, C), 50 µm; (A subpanels, B, Ci), 10 µm.

Morphological similarities with cytonemes and TNTs, led us to test whether EP bridges can act as conduits for intercellular communication. Time-lapse light microscopy showed a bleb-like structure, possibly a vesicle, moving through an EP bridge and into an EP island (Figure 3A). Live-cell imaging of lysosomes (Figure 3B) and the presence of a Golgi apparatus structural protein within vesicle-like structures in EP bridges (Figure 3C and Figure S7A) also supports the notion that these structures mediate transfer of cellular components between cells; however, these observations are not definitive proof that EP bridges transfer cellular cargo from one cell to another. In contrast to TNTs which can transport material between cells within minutes due to the relatively short distances TNTs span [5], [8], [9], the longer lengths of EP bridges present a technical issue for imaging the transfer of cellular components in live cells as longer lengths require longer exposure to fluorescent phototoxicity. Lysosomes were indeed observed moving through EP bridges for over an hour before movement was no longer detected. Also, the rate of component transfer may be faster in TNTs compared to EP bridges further complicating live cell imaging experiments. Future studies will be needed to provide definitive evidence EP bridges facilitate intercellular communication and material transport between cells.

**Figure 4:**
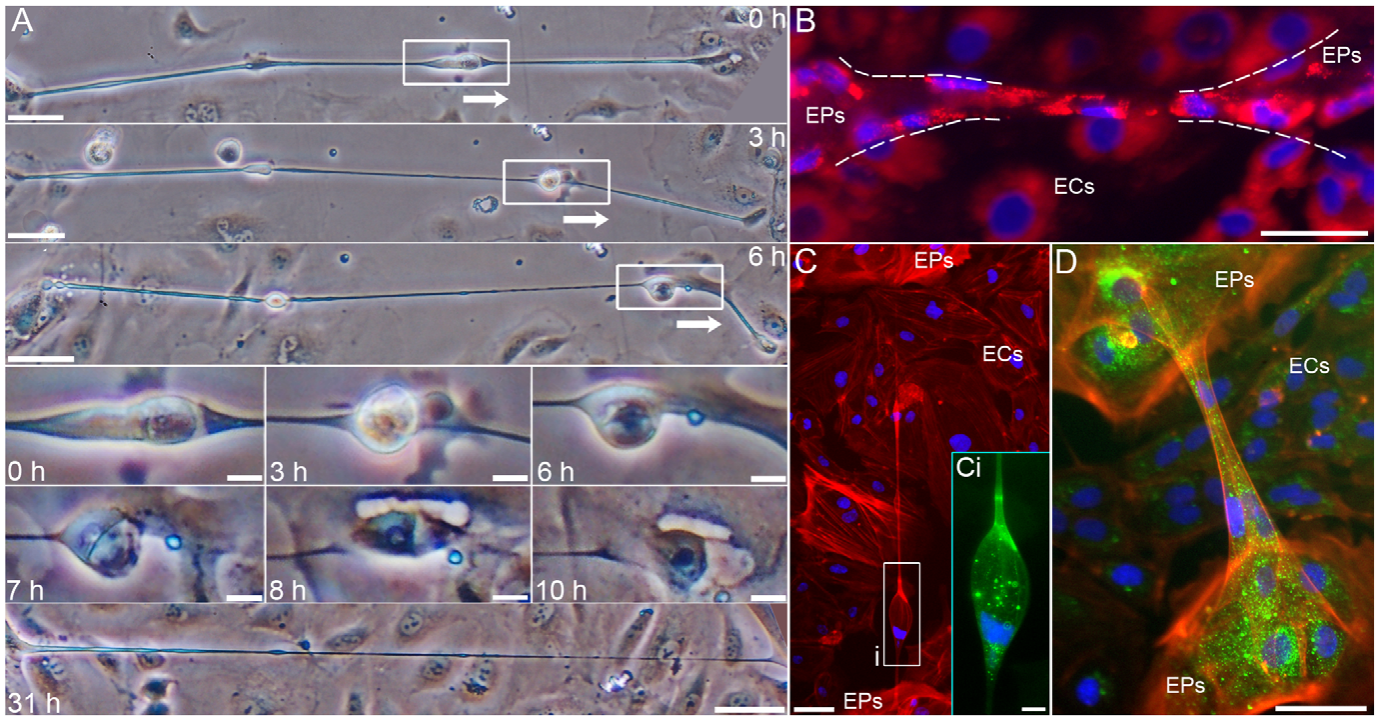
Cell migration via EP bridges. **A:** An individual cell migrating over 10 h through an EP bridge that connects EP islands (not a connection between only two cells). The cell expanded the elastic structure of the EP bridge, which returned to original size after the cell passed (0–6 h). The migrating cell exited the EP bridge through an elastic sheath and migrated into the EP island (7–10 h). EP bridge length remained relatively stable over 31 h as ECs grew underneath the bridge. The EP bridge measured 623 µm at 0 h and 562 µm at 31 h. **B:** Lysosome (red) and nucleus (blue) staining in EPs/ECs show nuclei co-localized with lysosomes in an EP bridge connecting two EP islands. **C–D:** F-actin (red), Golgi marker (green), and nucleus (blue) immunostaining show co-localization of nuclei and Golgi suggesting an individual cell expanded the EP bridge structure (C-Ci), while multiple cells were within another EP bridge (D). Scale bars: (A 0 h and 31 h, B, C, D), 50 µm; (A subpanels, Ci), 10 µm.

To date, cytonemes and TNTs have not yet been shown to transport genetic material, let alone cells; therefore, the existence of nuclei within a high percentage of EP bridges raised the question if genetic material was being transported between cells and/or cells were migrating between EP islands. Time-lapse light microscopy showed what appeared to be entire cells migrating through EP bridges (Figure 4A and Figures S8 to S9 with corresponding Movies S2 and S3, respectively). The migrating cells seemed to expand an elastic, tubular morphology of EP bridges which returned to original size as the cells migrated through the bridges. The possibility does exist that nuclei alone are being transported between EPs; however, in support of entire cells, and not just nuclei, migrating through EP bridges, immunofluorescent imaging showed co-localization of all nuclei with other cellular markers: lysosomes or Golgi apparatuses (Figure 4, B to D and Figure S7B). Also, the presence of the marker for non-mucus, secretory Clara cells within some EP bridges further supported the notion that specific EP bridges act as conduits for the movement of cells between EP islands (Figure S10). Further studies are needed to determine the specific types of cells moving within all EP bridges, but based on the composition of EP islands the most likely cell types migrating via these conduits are Clara cells and undifferentiated, transitional EPs. Overall these findings implied that two distinct connections between EP islands existed: the first being simple, tubular connections possibly mediating transport of cellular material between two cells (type I); and the second being complex, tubular connections facilitating migration of entire cells between multi-cellular EP islands (type II).

**Figure 5:**
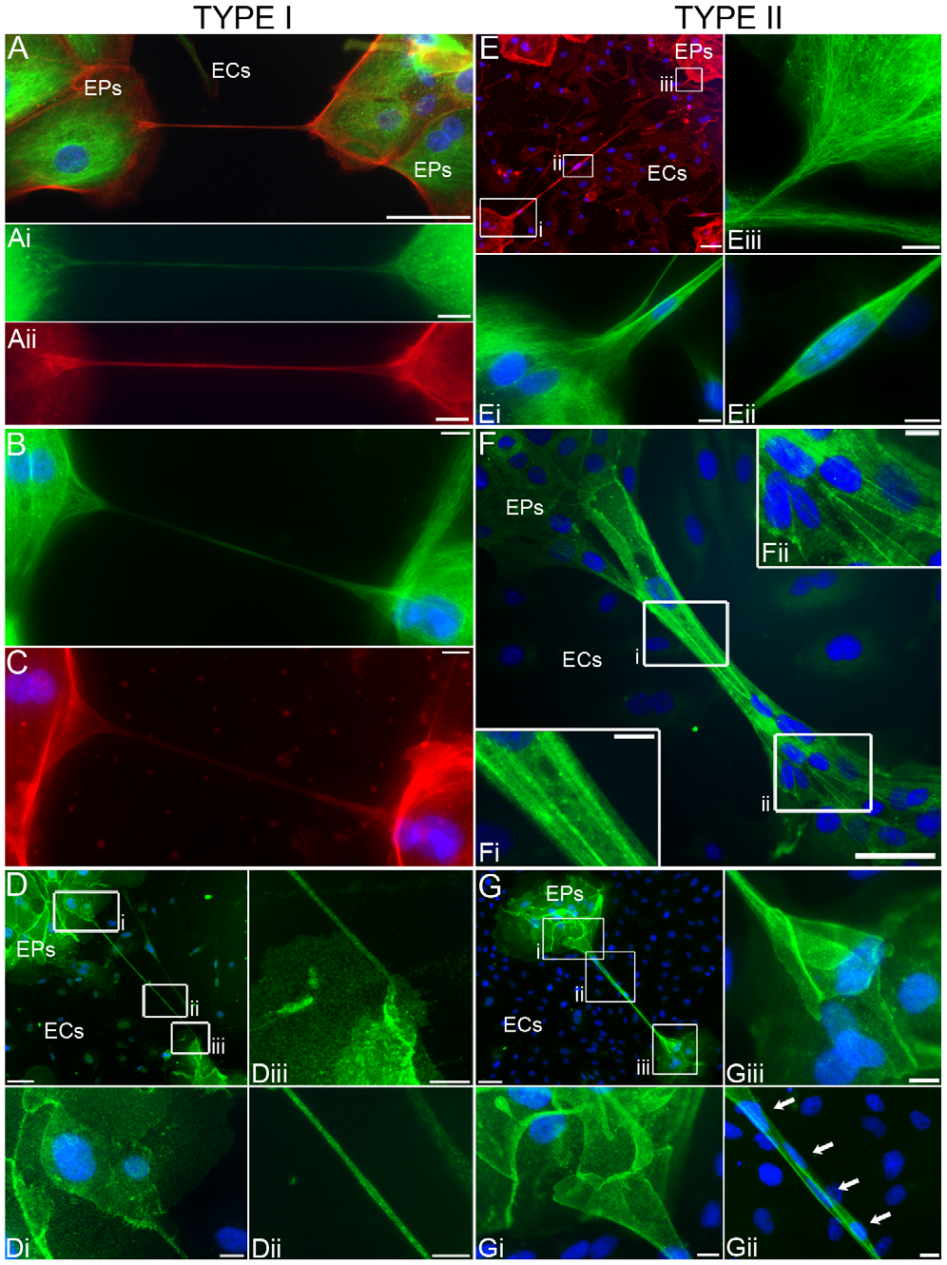
Characterization of types I and II EP bridge structures. **A–C:** Type I EP bridges connect a single cell from one EP island and a single cell from another EP island. (A) Color composite and color separation (Ai-Aii) of F-actin (red), microtubule (green), and nucleus (blue) staining showed an EP bridge connecting a single cell from one EP island to a single cell from another EP island. (B–C) Another type I EP bridge distinctly shows a connection between two cells from different EP islands. **D:** Nucleus (blue) and E-cadherin (green) immunostaining of a type I EP bridge extending 376 µm over ECs connecting 2 EPs. **E:** F-actin (red), nucleus (blue), and microtubule (green) immunostaining displayed F-actin (E) and microtubule architecture (Ei-Eiii) of a 570 µm type II EP bridge containing a nucleus at the lower end (Ei) and in the middle (Eii) of the conduit. **F:** Nucleus (blue) and E-cadherin (green) immunostaining showed a type II EP bridge as a continuous multi-cellular structure between EP islands. **G:** A type II EP bridge immunostained for E-cadherin (green) and nuclei (blue) structured with complex connections (Gi, Giii) between EP islands contained 4 nuclei (Gii, arrows indicate nuclei). Terminus of the EP bridge (Giii) resembled an elastic sheath similar to the structure observed in Fig. 4A: 7–10 h. Scale bars: (A, D–G), 50 µm; (Ai-Aii, B, C, Di-Diii, Ei-Eiii, Fi-Fii, Gi-Giii), 10 µm.

Structural characterization further elucidated the distinction between EP bridges. Immunostaining for F-actin, microtubules, and the epithelial-specific, transmembrane protein E-cadherin showed type I EP bridge connections were between individual cells (Figure 5, A to D). These F-actin and microtubule composed membrane tunnels appear to confer cytoplasmic connectivity between two cells similar to TNT structure. In contrast to type I EP bridges and TNTs, type II EP bridges possessed far more structural variation and complexity. Type II EP bridges ranged from thin, continuous, membranous conduits capable of allowing individual cells to migrate between EP islands (Figure 5E) to wide, continuous, membranous conduits capable of allowing multiple cells to migrate between EP islands (Figure 5F). In other type II EP bridges, cells were observed migrating out of elastic sheaths connected to EP islands (Figure 4A , 7 h to 10 h image panels). These sheath-like openings possess distinct E-cadherin borders suggesting these complex structures are derived from multiple EPs (Figure 5G). The distinct mechanisms involved in the dynamics of material transport and cell migration require future examination. Although one may hypothesize the F-actin and microtubule networks of EP bridges aid in facilitating the possible transport of cellular material between cells in type I bridges, while in type II bridges cell migration may be mediated by a combination of the distinct cytoskeletal networks within both the EP bridges and the migrating cells.

**Figure 6:**
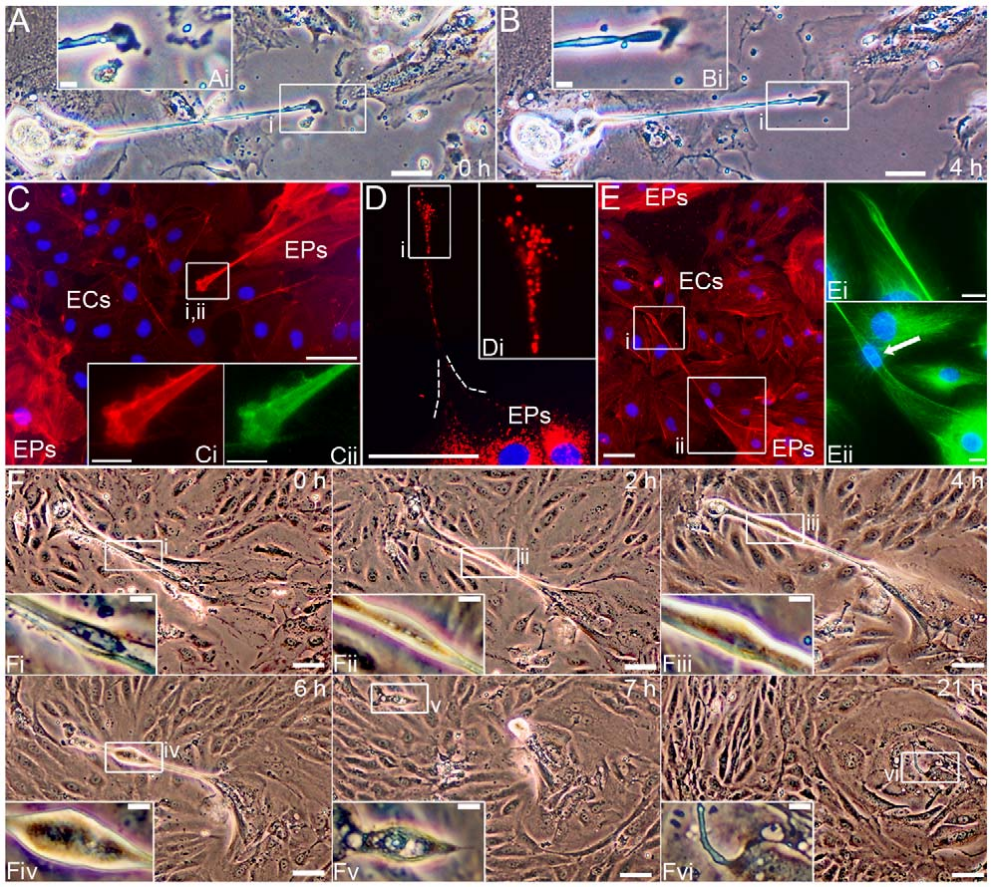
EP bridge formation via filopodia-like extensions. **A–B:** Time lapse light microscopy of EP islands connecting via a filopodia-like extension over 4 h in EPs/ECs. **C:** F-actin (red), nucleus (blue), and acetylated α-tubulin (green) immunostaining of filopodia-like structure showed F-actin (C-Ci) and microtubule architecture (Cii) extending from an EP island toward another EP island. **D:** Lysosome (red) staining throughout a filopodia-like extension. **E:** A nucleus extended into the F-actin (red) and microtubule (green, arrow indicates nucleus) extension before a connection could be established with another EP island. **F:** Retraction of a type II EP bridge after EPs in a smaller EP island migrated to a larger EP island over 7 h with only remnants of the EP bridge left after 21 h. Scale bars: (A–F), 50 µm; (Ai, Bi, Ci-Cii, Di, Ei-Eii, Fi-Fvi), 10 µm.

The formation of EP bridges was observed in two distinct manners. The first form involved filopodia-like extensions (Figure 6). Typical filopodia are F-actin-based cell extensions involved in sensory and exploratory functions of the cellular environment and participate in wound healing, invasion, chemotaxis, and cell-to-cell signaling [19]. This manner of EP bridge formation was similar to a form of TNT formation [12], [20] where filopodia-like extensions project from one cell making contact with a neighboring cell and allowing the TNT to be remodeled into a bridge (Figure 6, A and B ; and Movie S4). The second form of EP bridge formation involved an individual cell moving away from one EP island and connecting with another EP island while maintaining a direct physical connection with the first EP island (Movies S5 and S6). This second mode of bridge formation is also observed in TNTs where cell to cell connections elongate as the two cells move apart [12], [20]. EP extensions were composed of both F-actin and microtubules, varied in diameter, and could extend for hundreds of microns by moving over the substratum composed of other cell types (Figure 6C and Figure S11). Within these EP projections lysosomes (Figure 6D) and/or nuclei (Figure 6E) were present, suggesting EP bridge precursors may initiate cellular material transport and cell migration before connections between EPs or EP islands are established. The variable thickness of EP filopodia-like projections and the presence of cells within some of these structures suggest there may be distinct EP bridge precursors for type I and type II bridges, but more characterization will be needed to determine if there is a true distinction. The mechanisms directing filopodia-like extensions or elongation of cellular connections to form EP bridges are also unclear, but chemotactic guidance may play a role. During *Drosophila* development for instance, tracheal cells extend filopodia-like projections to cells expressing fibroblast growth factor [21]. In our culture system, once connections between EP islands are established intercellular signaling may take over as the dominant mechanism of EP bridge fate. EP bridge destruction could occur via retraction of the conduit (Movie S7) and in some instances occurred after most EPs in a smaller EP island migrated to a larger EP island via a type II EP bridge (Figure 6F). These observations support possible roles for chemotaxis, direct intercellular communication, and cell migration in determining EP bridge formation and fate.

How actual lumens form within EP bridges remains unclear. A recent study of TNTs showed that the octameric protein exocyst and the small GTPase RalA interact with the protein M-Sec. These protein interactions in one cell may play a role in protruding the membrane of another cell through actin remodeling and supplementation of membrane components to form a TNT [22]. Similar molecular interactions may be involved in the formation of tubular conduits leading to cytoplasmic connectivity between two cells in type I EP bridges. Lumen formation in type II EP bridges, however, would seem to require extensively more molecular and morphological changes to facilitate connections between multi-cellular EP islands and allow entire cells to migrate through these conduits. EP tube morphogenesis via cell or cord hollowing may mediate these changes allowing type II EP bridge lumen formation [23]. Cell hollowing involves a lumen forming within the cytoplasm of a single cell that spans the length of the entire cell and opens to the external environment at both ends. In the vertebrate microvascular system, capillaries are formed through cell hollowing of individual cells or chains of cells to create an intracellular lumen [24], while EPs composing secondary branches of the *Drosophila* tracheal system form unicellular tubes possibly through cell hollowing as well [25]. These secondary branch EPs also extend long cytoplasmic projections in which a narrow lumen forms and creates a seamless connection with the intracellular lumen of the secondary branch. Type II EP bridges with thin and continuous conduits (Figure 5E) may be the product of individual cells or chains of cells undergoing cell hollowing. Type II EP bridges that are wider and composed of multiple cells (Figure 5, F and G) might also be formed via cell hollowing or instead through the process of cord hollowing. In cord hollowing, a lumen is formed de novo between cells assembling in a thin cylindrical cord with no loss of cells in the process. Cord hollowing has been reported in Drosophila heart [26], *Caenorhabditis elegans* intestine [27], and Madin-Darby canine kidney cultured cells [28]. In both processes of lumen formation vesicular structures known as vacuolar apical compartments may be involved [23], [29]. These specialized organelles are formed via plasma membrane endocytosis and fuse together to aid in intracellular lumen formation. The lumen openings of type II EP bridges require detailed characterization, but appear to be either embedded within or on the surface of the multi-cellular EP islands allowing cells within EP islands to migrate in and out of these conduits. Whether lumen formation in type II EP bridges occurs through cell hollowing, cord hollowing, or another process, the ability of EP cellular extensions to form these conduits may be through de novo biogenesis via filopodia-like extensions distinct from type I EP bridge extensions and/or morphological transition from type I EP bridges.

**Figure 7:**
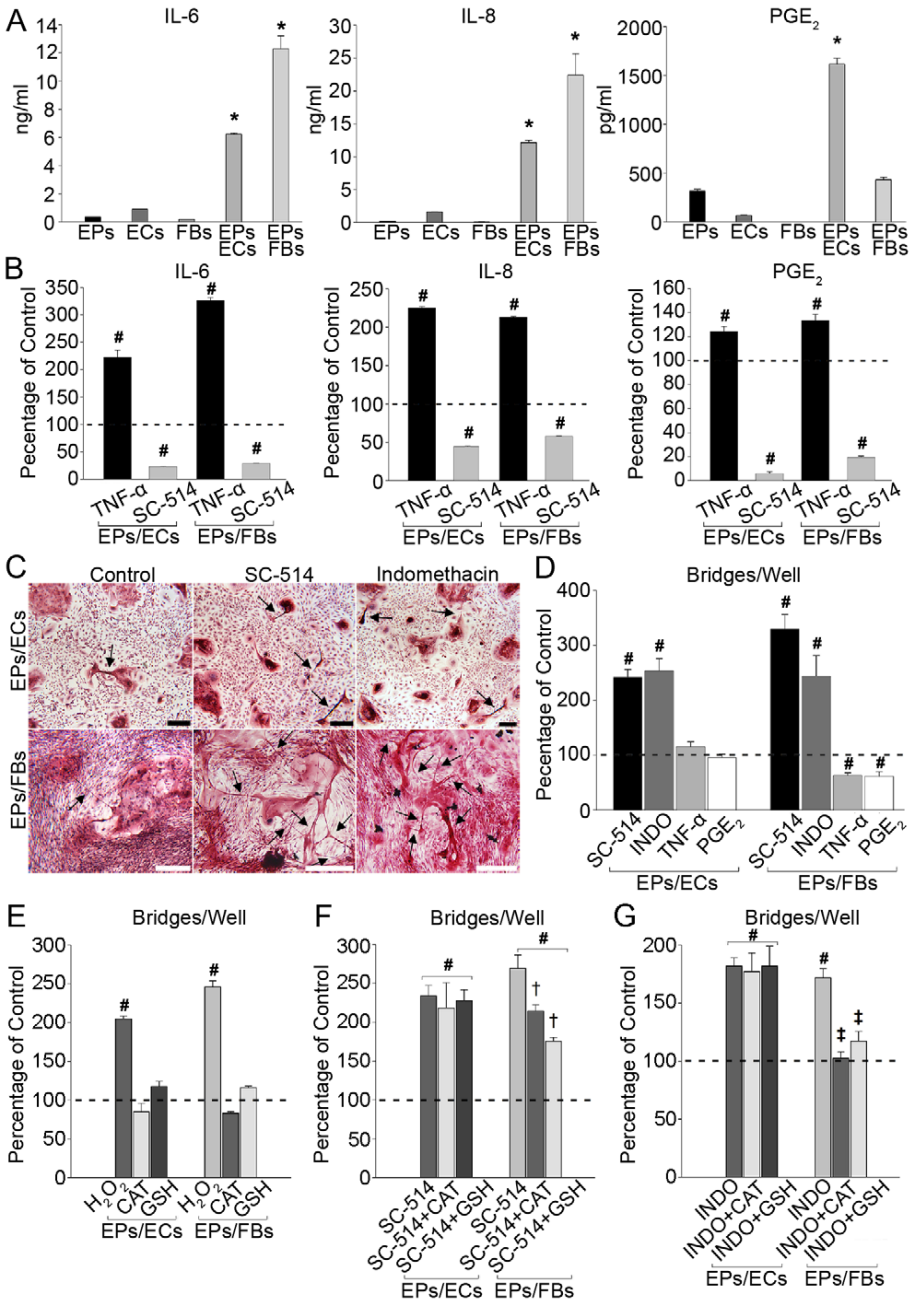
NF-κβ, COX, and ROS involvement in EP bridge formation. **A:** IL-6, IL-8, and PGE_2_ levels in 24 h conditioned medium show increased inflammation in co-cultures vs. monocultures. *p<0.05 vs. respective monocultures, n = 4−5. **B:** TNF-α (25 ng/ml), an NF-κβ activator, increased IL-6, IL-8, and PGE_2_ secretion after 48 h. SC-514 (100 µM), an NF-κβ inhibitor, decreased secretion of all three proteins after 48 h in EPs/ECs and EPs/FBs. #p<0.05 vs. control, n = 4−5. **C–D:** SC-514 or indomethacin (INDO, 50 µM), a COX pathway inhibitor, caused ∼2–3 fold increases in EP bridges, arrows denote EP bridges, for both EPs/ECs and EPs/FBs after 48 h, n = 6−8. Scale bars, 250 µm. TNF-α or PGE_2_ (10 µM) decreased EP bridges in only EPs/FBs. #p<0.05 vs. control, n = 3−4. **E:** H_2_O_2_ (250 µM) increased EP bridges in co-cultures, while antioxidants, CAT and GSH, had no effect on control EP bridges after 48 h. Addition of CAT or GSH diminished stimulation of EP bridge formation caused by SC-514 or INDO only in EPs/FBs after 48 h. #p<0.05 vs. control; †p<0.05 vs. SC-514; ‡p<0.05 vs. INDO; n = 3−4. Error bars indicate SEM.

In an attempt to understand biochemical pathways regulating EP bridge form and function, we looked at molecules involved in inflammatory airway diseases, such as asthma and chronic obstructive pulmonary disorder. In these diseases the EP lining undergoes extensive morphological remodeling due in part to the upregulation of various inflammatory molecules, such as interleukin (IL)-6, IL-8, and prostaglandin (PG) E_2_ [15], [30]. Although the remodeling in asthma or chronic obstructive pulmonary disorder is not the same as the alterations EPs undergo in forming EP bridges, we reasoned that these processes may share regulatory pathways that mediate the observed morphological changes. In EPs/ECs and EPs/FBs, IL-6 and IL-8 were significantly increased compared to monocultures (Figure 7A), suggesting interactions between EPs and other cell types activate an inflammatory response in vitro resulting in EP remodeling that may play a role in EP bridge formation. Also, a significant increase in PGE_2_ was measured only in EPs/ECs implying the COX pathway, which metabolizes arachidonic acid into PGs [31], is upregulated when EPs interact with ECs but not with FBs. The increase in IL-6 and IL-8, and divergent levels of PGE_2_ in co-cultures led us to test whether EP bridge formation involved the transcription factor NF-κβ, which regulates these three proteins along with many other cell migration and inflammatory molecules [30], [32]. Tumor necrosis factor (TNF)-α, an activator of NF-κβ, significantly increased IL-6, IL-8, and PGE_2_ levels indicating NF-κβ was not maximally stimulated in co-cultures; however, blocking NF-κβ activation in control co-cultures reduced levels of all three proteins ∼50% to 95% suggesting morphological and biochemical interactions between EPs and other cell types activated NF-κβ resulting in the observed inflammatory response in vitro (Figure 7B). Interestingly, blocking NF-κβ activity increased EP bridge numbers ∼2 to 3 fold in co-cultures (Figure 7, C and D), which countered our notion that increases in inflammatory molecules would stimulate EP remodeling which in turn would increase EP bridge numbers. Instead these results implied that inflammation may impede the formation of EP bridges. Determining the existence of EP bridges in airway inflammatory-diseased cells and possibly manipulating bridge formation via NF-κβ and other inflammatory pathways will be of great interest.

Because NF-κβ regulates a variety of proteins, blocking any of which may have affected EP bridge formation, we focused on the differences in EP bridge numbers and PGE_2_ levels between EPs/ECs and EPs/FBs where higher EP bridge numbers inversely correlated with lower PGE_2_ levels (Figure S12). In the airway, PGE_2_ can be pro- or anti-inflammatory and regulates cell migration and proliferation [33], [34]. Reducing PGE_2_ by blocking COX activity, both constitutive (COX-1) and inducible (COX-2) isoforms, via indomethacin (INDO) caused EP bridge formation to increase ∼2 fold for both co-cultures (Figure 7, C and D). Either stimulating COX activity with TNF-α or adding exogenous PGE_2_ to co-cultures diminished EP bridge numbers ∼40% in EPs/FBs where endogenous PGE_2_ was low, while EP bridge numbers were unaffected in EPs/ECs where endogenous PGE_2_ was high (Figure 7, A and D), suggesting that up to certain saturation levels in co-cultures PGE_2_ impedes EP bridge formation. Overall, a partial role for PGE_2_ in EP bridge regulation appears evident, but roles for other pathways and products remain to be elucidated.

**Figure 8:**
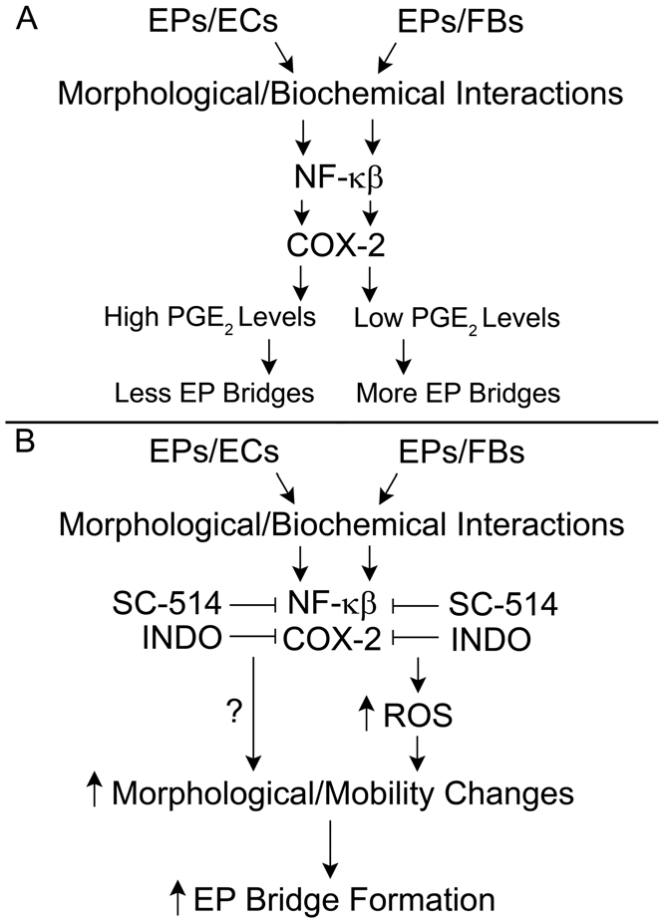
Morphological and biochemical interactions regulate EP bridge formation through mediation of inflammation. **A:** Cellular and biochemical interactions between EPs and ECs or FBs may differentially regulate the NF-κβ and COX inflammatory pathways, producing higher levels of PGE_2_ in EPs/ECs compared to EPs/FBs. PGE_2_ impedes EP bridge formation and fewer EP bridges are observed in EPs/ECs compared to EPs/FBs. **B:** Pharmacologic blockade of these inflammatory pathways with SC-514 (an NF-κβ inhibitor) or indomethacin (INDO, a COX inhibitor) increases morphological and mobility changes, leading to increased EP bridge formation in both co-cultures. Inhibition of both NF-κβ and COX pathways in only EPs/FBs may stimulate ROS production and in that manner increase EP bridge formation.

In various cell types, NF-κB and COX-2 are regulated by ROS, which modulate cell functions such as inflammation, cell growth, and motility [35], [36]. Occurring as byproducts of reactions between molecular oxygen with mitochondria, peroxisomes, and other cellular elements, ROS include oxygen radicals (e.g. superoxide) and non-radicals (e.g. hydrogen peroxide, H_2_O_2_) which are converted into radicals and/or are oxidizing agents [37]. Exposure to antioxidants, catalase (CAT) or reduced L-glutathione (GSH), did not affect EP bridge formation in control co-cultures suggesting ROS are not involved in EP bridge regulation under control conditions. Yet exogenous H_2_O_2_ addition did increase EP bridge numbers ∼2 fold in co-cultures implying ROS can stimulate EP bridge formation (Figure 7E). In previous studies, NF-κB deficiency in mouse embryo fibroblast cells and a human EP cell line caused elevation of H_2_O_2_ which mediated morphological and mobility changes including extended cellular protrusions [38], [39]. In our system, the addition of antioxidants in NF-κB or COX inhibited co-cultures caused divergent responses in EP bridge formation where decreased ROS levels resulted in significantly diminished EP bridge numbers only in EPs/FBs (Figure 7, F and G). These findings suggest NF-κB or COX blockade increased ROS levels which played a role in increased EP bridge formation in EPs/FBs. Antioxidant addition in only EPs/FBs partially blocked NF-κB inhibition-dependent increases in EP bridges, but completely abrogated COX inhibition-dependent increases, suggesting ROS regulation is only partially controlled through NF-κB possibly through the regulation of the COX pathway, while the COX pathway appears to fully regulate ROS levels in EPs/FBs. Supporting these findings, a previous study showed renal mesangial cells over expressing COX-2 possessed decreased ROS concentrations suggesting regulation of the cellular redox system was COX-2 dependent [40]. In EPs/ECs, the mechanism controlling EP bridge increases after NF-κB or COX inhibition requires further study. Overall, a model of EP bridge regulation begins to emerge where morphological and biochemical interactions differentially modulate inflammatory pathways which in turn affect EP bridge formation (Figure 8A). Abrogation of these inflammatory pathways results in increased morphological and mobility changes stimulating EP bridge formation (Figure 8B). These findings support the notion EP bridges are a normal physiological characteristic of EPs, at least in vitro, which is impeded by inflammation.

Cellular extensions are associated with various physiological processes including embryogenesis, tumorigenesis, and the immune response [12], [41]. In lung development, EPs participate in branching morphogenesis where the tubular epithelial network gradually branches into thinner conduits and in concert with migration of lung ECs and FBs [29], [42]. EP bridges may enable coordinated cell coalescence, signaling, and migration. Also, the culturing of EPs, particularly with other cell types, may activate dormant, developmental pathways that propel an inherent plasticity to form tubular structures, albeit with a function that transcends oxygen perfusion, and suggests EPs in all human beings have an inherent ability to form these structures. The possible transport of cellular components and migration of entire cells via EP bridges is intriguing with the latter not seen in other forms of cellular extensions in the animal or plant kingdoms to date. The questions of in vivo counterparts to and the physiological relevance of EP bridges remain to be answered. However, if EP bridges are important for intercellular communication and/or cell migration, further functional and molecular understanding of these structures may provide beneficial insights into various biological processes.

## Materials and Methods

### Cell Culture

All human cells used were primary cells with 3 to 5 donor populations used either separately or pooled for each cell type. Cells and medium were obtained from Lonza, used between passages 2 to 8, and cultured at 5% CO_2_, 37°C. EPs cryopreserved with retinoic acid were grown in BEGM supplemented with 0.4% bovine pituitary extract; 0.1% hydrocortisone; 0.1% human EGF; 0.1% epinephrine; 0.1% transferring; 0.1% insulin; 0.1% retinoic acid; 0.1% triiodothyronine; 0.1% gentamicin, amphotericin-B (GA-1000); and 20 units/ml penicillin-streptomycin. ECs and FBs were grown in EGM-2 supplemented with 10% FBS; 0.4% human epidermal growth factor; 0.04% hydrocortisone; 0.1% R3-insulin-like growth factor-1; 0.1% vascular endothelial growth factor; 0.1% human FGF; 0.1% ascorbic acid; 0.1% heparin; 0.1% GA-1000; and 20 units/ml penicillin-streptomycin.

Cells were grown on gelatin-coated (10 mg/ml, Sigma) tissue culture polystyrene 100 mm plates, 6-well plates, or 2-chamber culture slides (Becton-Dickinson). Co-cultures were created by seeding EPs with ECs or FBs at a ratio of 2∶1 (EPs∶ECs or FBs). Cell types were seeded separately on the same culture area, the second cell type immediately after the first, then mixed thoroughly together at a pre-confluent level of 75,000 total cells per well in 2-chamber culture slides or 150,000 total cells per well for 6-well plates. A 1∶1 mixture of BEGM∶EGM-2 with a total of 5% FBS was used for co-cultures and for cultures of EPs when comparing EP bridge formation in all experiments, unless otherwise stated.

Air-liquid interfaces were established on 0.4 µm membranes in 12 mm transwell inserts (Costar) and coated with human placenta collagen IV (Sigma) overnight. EPs were seeded at 150,000 cells on the apical surface of each membrane. BEGM was added to the apical and basal areas of the insert allowing attachment of EPs to the membrane for 24 h. The medium was then removed and the insert inverted on a dry culture plate allowing 15,000 FBs to be seeded on the basolateral side of the membrane for 45 min at 5% CO_2_, 37°C. The insert was then returned to the well and an air-liquid interface established with a 1∶1 mixture of BEGM∶DMEM (low glucose) with 5% FBS used as the basolateral medium. After 3 weeks, the cells were fixed with 4% paraformaldehyde for microscopic analysis. For all culture conditions medium was changed every 2 to 3 days.

### Biosecretory Characterization

After 24 or 48 h in cell culture, cell conditioned medium with 5% FBS was collected and analyzed using commercially available ELISA kits according to the manufacturers' instructions. Levels of PGE_2_ (Cayman Chemical), and IL-6 and IL-8 (Invitrogen) were measured for EPs, ECs, and FBs alone or in co-cultures. To obtain the baseline/background level of each soluble factor in the medium without cells, fresh medium with 5% FBS was incubated for 24 or 48 h without cells and measured in parallel with medium exposed to cells. This baseline/background level in the medium without cells was subtracted from the levels in medium exposed to cells to obtain the actual levels of soluble factors produced by the cells alone.

### COX, NF-κβ, and ROS Stimulation and Inhibition

PGE_2_ was obtained from Cayman Chemical; catalase, indomethacin, TNF-α, and H_2_O_2_ were obtained from Sigma; while SC-514, an NF-κβ inhibitor [43], and reduced L-glutathione were obtained from Calbiochem. Cultures were grown to 95–100% confluence on 2-well culture slides before addition of any stimulator and/or inhibitor. Fresh BEGM:EGM-2 with 5% FBS was added with the addition of each inhibitor and/or stimulator, and collected after 48 h. Control samples contained the same amount of vehicle in the medium as experimental conditions.

### Cell Imaging

Time-lapse light microscopy of cells grown on 6-well plates was performed with an inverted microscope (Nikon Diaphot) and digital camera (Nikon D50) using 4x and 10x lenses over specified time periods. For immunofluorescence, cells were fixed for 20 min. with 4% paraformaldehyde. Cells were exposed to 0.2 M glycine for 10 min and incubated with 0.2% triton X-100 in phosphate buffered saline for 10 min. Goat serum (4%) in phosphate buffered saline with 1% bovine serum albumin was applied for 1 h at room temperature (RT). Primary antibodies were applied to the cells overnight at 4°C: Collagen IV (1∶50, AbCam); E-cadherin, Golgi marker, vimentin, CC10, MUC5AC, cytokeratin-5, smooth muscle α-actin (1∶50, Santa Cruz); CD31 (1∶50, Serotec); anti-acetylated α-tubulin (1∶50, Sigma). Secondary antibodies – alexa fluoro 488 and 594 (1∶50, Invitrogen) – were applied to the cells for 1 h at RT with or without rhodamine-phalloidin (1∶250, Invitrogen) for visualization of F-actin. Cells were mounted with VectaShield containing DAPI (Vector Labs). Image-iT™ LIVE Lysosomal and Nuclear Labeling Kit was used following the manufacturer's instructions (Invitrogen) for staining of lysosomes and nuclei. All images were obtained using a Zeiss LSM510 Laser scanning confocal microscope with a 25x lens or a Leica DMRA2 fluorescent microscope with 10x, 20x, 40x, and 100x lenses, and a Hamamatsu C4742-95 digital camera and MetaMorph software. ImageJ 1.42a software was used to generate 3D images from confocal image stacks of 50 to 100 consecutive 0.5 µm z-slices.

Masson Trichrome straining (IMEB) was performed using Bouin's solution as a mordant overnight at RT. Cells were stained with Hematoxylin for 10 min, Biebrich's Scarlet Acid Fuchsin for 15 min, followed by Phosphotungstic/Phosphomolybdic Acid staining for 15 min. Aniline Blue stain was applied for 15 min. then cells were exposed to 1% acetic acid solution for 1 min, dehydrated through an ethanol series, cleared with xylenes for 5 min, and mounted with Cytoseal XYL (EMS). Photomicrographs were taken with a DC-70 digital camera (Olympus) on a Nikon Labphoto-2 microscope with 4x, 10x, 20x, and 40x lenses.

### Cell Morphological Analysis

Adobe Photoshop CS3 and ImageJ 1.42a software were used to characterize EP bridges by measuring the length, diameter, number of nuclei in each EP bridge, and the total number of EP bridges in a 4 cm^2^ culture area (the size of one well in a 2-chamber culture slide).

### Statistical Analysis

Data are expressed as standard error of the mean. Statistica 6.0 software was used to perform statistical analysis. Student's t test analysis was performed for data between 2 groups. ANOVA followed by Fisher's least significant difference post hoc test to assess intergroup significance was used to evaluate data among >2 groups. Significance was considered as a value of p<0.05.

## Supporting Information

Figure S1 EP cysts and Clara cells within EP islands. A–D: Trichrome staining of EP islands in EP/ECs or EPs/FBs showed EP cysts form within EP islands. E: A cell specific marker (CC10, green) revealed non-mucus, secretory Clara cells partially compose EP islands in EPs/ECs. F: Composite of image E with immunostaining for CC10 (green), F-actin (red), and nucleus (blue) show other types of EPs also compose EP islands. Immunostaining for differentiated basal (Cytokeratin 5) and mucus secreting (MUC5AC) EPs was negative (data not shown) suggesting the remaining composition of EP islands is derived from undifferentiated, transitional EPs. Scale bars: 50 µm.
(5.45 MB TIF)

Figure S2 EP bridge expands over time. A–D: Time lapse light microscopy of an EP bridge expanding over 27 h in EPs/ECs (white bars are measurement points for EP bridge length). The EP bridge expanded from (A) 434 µm at 0 h to (B) 564 µm at 5 h to (C) 1.05 mm at 27 h. EPs expanded the elastic tubular structure of the EP bridge at 2 points (Ci-Cii). (D) Lower magnification image at 27 h of EP islands connected by the EP bridge (arrows indicate EP bridge). Scale bars: (A-Cii), 50 µm; (D), 100 µm.
(5.22 MB TIF)

Figure S3 Morphology of EP bridges and EP islands in co-cultures of EPs and FBs. A–B: Trichrome staining in EPs/FBs of EP islands surrounded by FBs. C: Nucleus (blue) and E-cadherin (green) immunostaining displayed segregation of EPs and FBs. D: Staining of F-actin (red) in EPs and FBs. E–F: Trichome staining of EP bridges between EP islands with (E-Ei) and without (F-Fiii) nuclei. Scale bars: (A), 500 µm; (B–F), 50 µm; (Ei, Fi-Fiii), 10 µm.
(6.16 MB TIF)

Figure S4 EP islands and EP bridges do not express the EMT marker α-smooth muscle actin. A–B: Nucleus (blue), α-smooth muscle actin (red, A), and E-cadherin (green, B) immunostaining showed only FBs expressed α-smooth muscle actin in EPs/FBs. C–H: EP islands (C–D) and EP bridges without (E–F) or with nuclei (G–H) did not express α-smooth muscle actin in EPs/ECs (arrow indicates EP bridge in panel F). Panels A, C, E, and G correspond to panels B, D, F, and H respectively. Scale bars: 50 µm.
(4.28 MB TIF)

Figure S5 EP islands and EP bridges do not express the EMT marker vimentin. A–C: F-actin (red), vimentin (green), and nucleus (blue) immunostaining show EP bridges without (A–B) and with nuclei (C) do not express vimentin. Scale bars: 50 µm.
(4.18 MB TIF)

Figure S6 EP bridges form in air-liquid interfaces. A–C: EPs were grown on the apical surface of collagen IV-coated membranes and exposed to air, while FBs were grown on the basolateral side of the same membrane and grown in 1∶1 mixture of BEGM:DMEM (low glucose) with 5% FBS for 3 weeks. EPs (A) on the apical surface and FBs (B) basolateral surface of the same membrane were immunostained for microtubules (green), F-actin (red), and nuclei (blue), while (C) EP bridge formation was observed even with EPs exposed to air. Scale bars: 50 µm.
(4.93 MB TIF)

Figure S7 Golgi apparatuses localize to vesicle-like structures and cells in EP bridges. A: F-actin (red), Golgi marker (green), and nucleus (blue) immunostaining show a vesicle-like structure positive for Golgi in an 801 µm EP bridge in EPs/ECs. No nuclei were present in the EP bridge (A-Ai) or vesicle-like structure (Aii) (arrows indicate vesicle-like structure position). B: F-actin (red), Golgi marker (green), and nucleus (blue) immunostaining show a cell within an EP bridge (arrows indicate cell position). Scale bars: (A-Ai, B), 50 µm; (Aii, Bi), 10 µm.
(4.15 MB TIF)

Figure S8 Cell migration through a type II EP bridge in EPs/ECs. A–C: Sequential images taken from Movie S2 show a cell in the middle of an EP bridge migrating towards an EP island in the top left of each image over 10 h. Panels Ai–Ci are magnified sections of A–C. Arrows indicate location of the cell within the EP bridge. Scale bars: 50 µm.
(4.42 MB TIF)

Figure S9 Cell migration through a type II EP bridge in EPs/ECs. A–D: Sequential images taken from Movie S3 show a cell within an EP bridge connected to an EP island on the left side migrating left to right toward the connected EP island on right side over 10 h. Panels Ai–Di are magnified sections of A–D. Arrows indicate location of the cell within the EP bridge. Scale bars: 50 µm.
(6.37 MB TIF)

Figure S10 Clara cell within a type II EP bridge. A: Immunostaining for CC10 (green) and nuclei (blue) shows a Clara cell within an EP bridge in EPs/ECs. B: Addition of F-actin (red) to color composite in image A shows EP bridge and EP island architecture. Scale bars: 50 µm.
(1.96 MB TIF)

Figure S11 Architecture of EP filopodia-like extensions. A–B: Magnified images of Figure 4E (A) and 4Eii (B) where F-actin (red), nucleus (blue), and microtubule (green) immunostaining displayed a nucleus extending into the F-actin (A) and microtubule (B) structure of the filopodia-like extension in EPs/ECs (arrows indicate nucleus). C: Color composite of F-actin (red) and microtubule (green) expression of an EP bridge precursor from one EP island extending toward another EP island at the leading edge (Ci). F-actin (Cii) and microtubule (Ciii) expression at the base of the EP extension (arrows indicate nuclei). D–E: Trichome staining in EPs/ECs showed an EP filopodia-like structure extending from one EP island to another EP island (panels E is a magnified image of panel D). F–G: Wide EP filopodia-like structure extending out from one EP island toward another EP island (panel G is a magnified image of panel F). Scale bars: 50 µm.
(4.85 MB TIF)

Figure S12 PGE2 expression affects EP bridges formation. EP bridge formation inversely correlates with PGE2 levels from 24 h conditioned media in co-cultures. Closed circles are EPs/FBs, open circles are EPs/ECs.
(0.47 MB TIF)

Movie S1 EP bridge brings EP islands together over time. Two EP islands connected by an EP bridge migrated towards each other, which reduced EP bridge length until the EP bridge disappeared as the two EP islands became one EP island in EPs/ECs over 7 h. Each frame was taken using a Nikon Diaphot inverted microscope with a 10x phase lens. Time point for each frame is specified in the video. Scale bars: 50 µm.
(0.76 MB MOV)

Movie S2 Cell migration through a type II EP bridge in EPs/ECs. An epithelial cell in the middle of an EP bridge migrates toward the EP island in the top left over 10 h. Images were taken using a Nikon Diaphot inverted microscope with a 10x phase lens. Time point for each frame is specified in the video. Scale bars: 50 µm.
(1.37 MB MOV)

Movie S3 Cell migration through a type II EP bridge in EPs/ECs. An epithelial cell within an EP bridge connected to the EP island on left side migrates left to right toward the connected EP island on the right side over 13 h. Once the cell reached the right side EP island the connection of the EP bridge with the left side EP island broke. Each frame was taken using a Nikon Diaphot inverted microscope with a 10x phase lens. Time point for each frame is specified in the video. Scale bars: 50 µm.
(3.57 MB MOV)

Movie S4 Filopodia-like extension forms an EP bridge in EPs/ECs. A filopodia-like extension projects out from an epithelial cell and connects to an EP island over 5.5 h. Each frame was taken using a Nikon Diaphot inverted microscope with a 10x phase lens. Time point for each frame is specified in the video. Scale bars: 50 µm.
(0.63 MB MOV)

Movie S5 Filopodia-like extension with a cell at the leading edge forms an EP bridge in EPs/ECs. An epithelial cell at the leading edge of a filopodia-like extension migrates toward the top right connecting with an EP island over 6 h. Each frame was taken using a Nikon Diaphot inverted microscope with a 10x phase lens. Time point for each frame is specified in the video. Scale bars: 50 µm.
(2.05 MB MOV)

Movie S6 Filopodia-like extension with a cell at the leading edge forms EP an bridge in EPs/ECs. An epithelial cell at the leading edge of a filopodia-like extension migrates right to left connecting with an EP island over 6 h. Each frame was taken using a Nikon Diaphot inverted microscope with a 10x phase lens. Time point for each frame is specified in the video. Scale bars: 50 µm.
(0.89 MB MOV)

Movie S7 Retraction of an EP bridge connecting EP islands. An EP bridge retracts over 3 h in EPs/ECs. Each frame was taken using a Nikon Diaphot inverted microscope with a 4x phase lens. Time point for each frame is specified in the video. Scale bars: 50 µm.
(0.48 MB MOV)
