# Supplementary figures and images for: Tubular Bridges for Bronchial Epithelial Cell Migration and Communication
Source: PLoS One. 2010 Jan 28;5(1):e8930. doi: 10.1371/journal.pone.0008930 (PMC2812493; doi:10.1371/journal.pone.0008930)

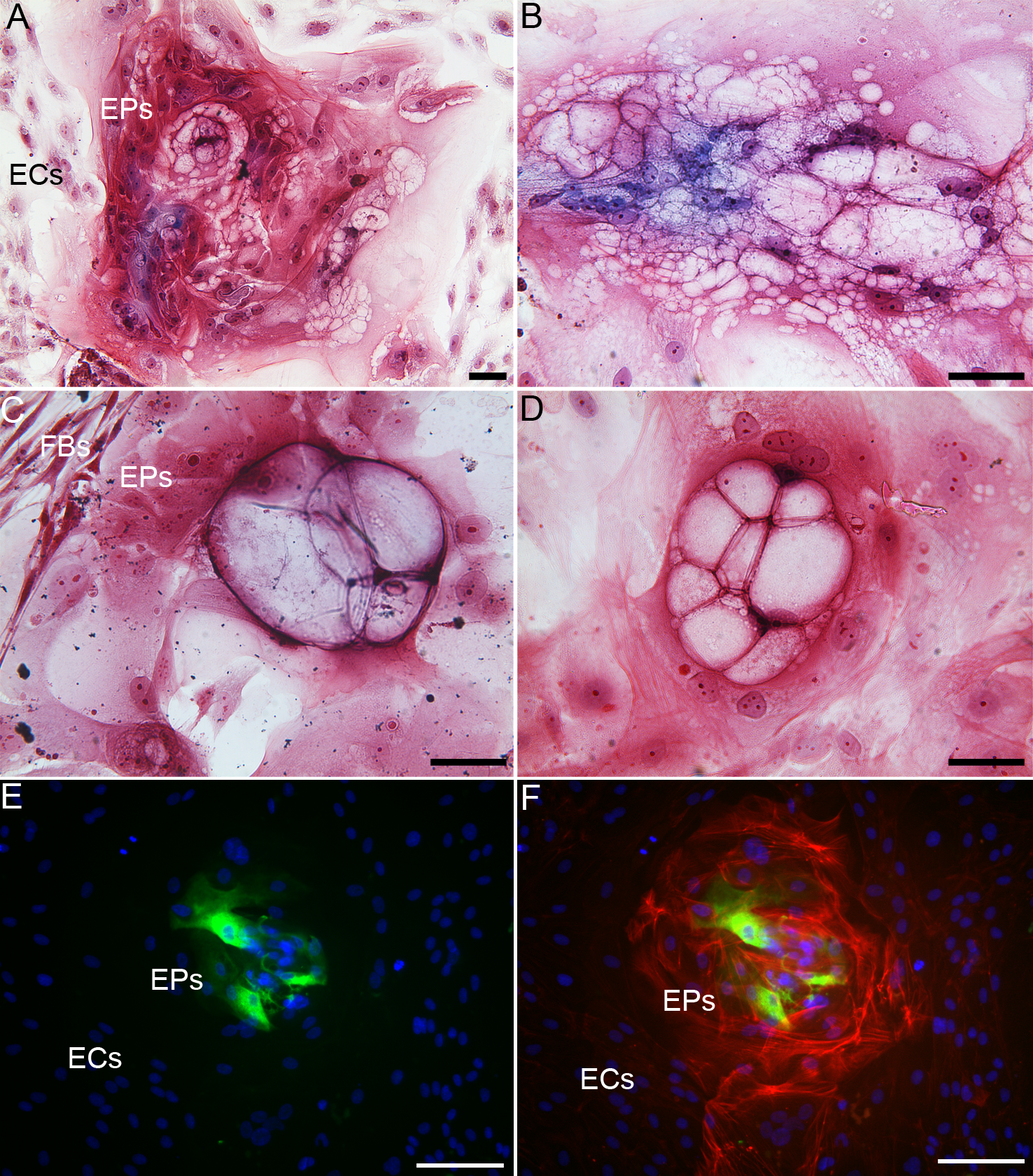

Supplement: Figure S1 — EP cysts and Clara cells within EP islands. A–D: Trichrome staining of EP islands in EP/ECs or EPs/FBs showed EP cysts form within EP islands. E: A cell specific marker (CC10, green) revealed non-mucus, secretory Clara cells partially compose EP islands in EPs/ECs. F: Composite of image E with immunostaining for CC10 (green), F-actin (red), and nucleus (blue) show other types of EPs also compose EP islands. Immunostaining for differentiated basal (Cytokeratin 5) and mucus secreting (MUC5AC) EPs was negative (data not shown) suggesting the remaining composition of EP islands is derived from undifferentiated, transitional EPs. Scale bars: 50 µm. (5.45 MB TIF) [file pone.0008930.s001.tif]

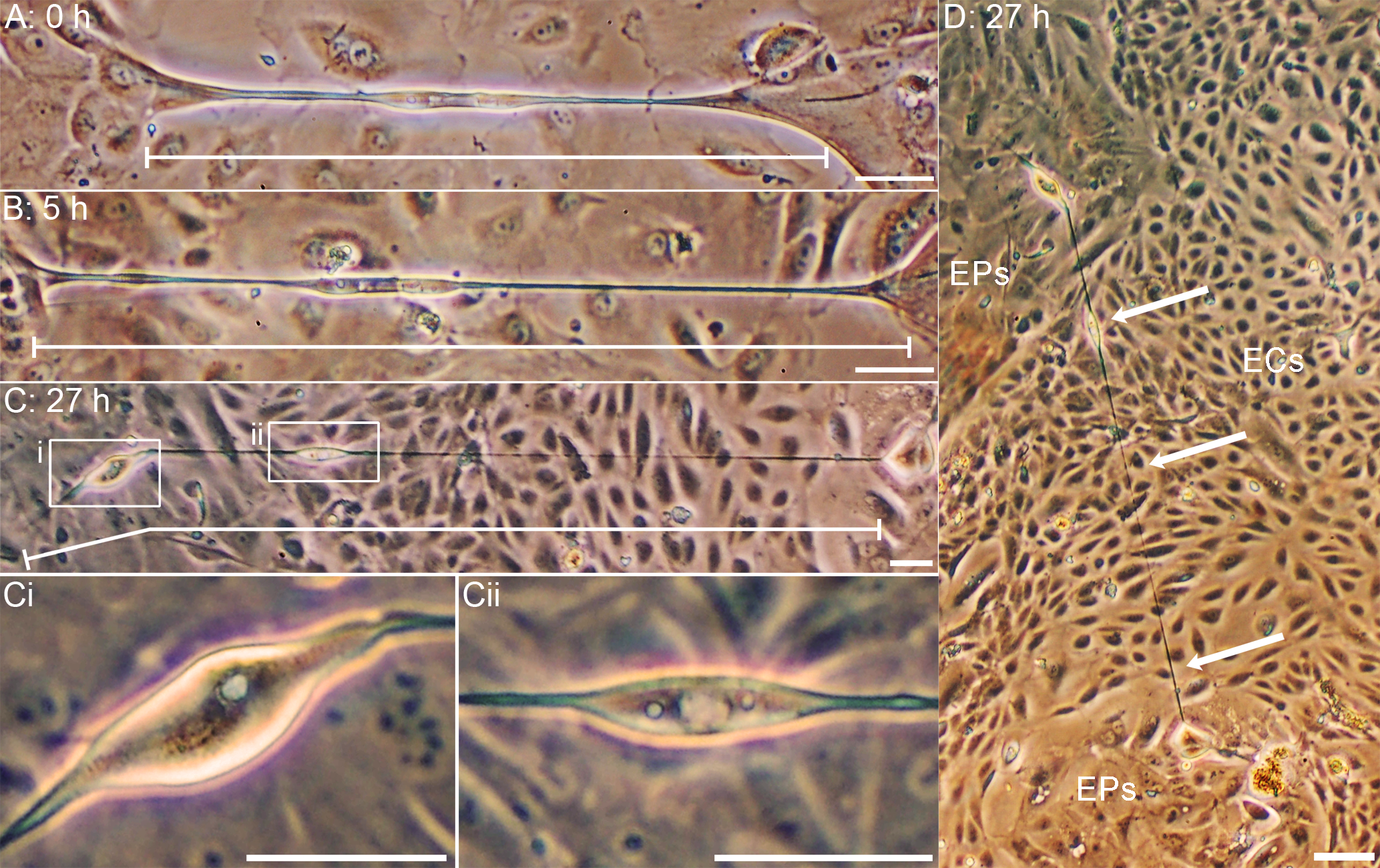

Supplement: Figure S2 — EP bridge expands over time. A–D: Time lapse light microscopy of an EP bridge expanding over 27 h in EPs/ECs (white bars are measurement points for EP bridge length). The EP bridge expanded from (A) 434 µm at 0 h to (B) 564 µm at 5 h to (C) 1.05 mm at 27 h. EPs expanded the elastic tubular structure of the EP bridge at 2 points (Ci-Cii). (D) Lower magnification image at 27 h of EP islands connected by the EP bridge (arrows indicate EP bridge). Scale bars: (A-Cii), 50 µm; (D), 100 µm. (5.22 MB TIF) [file pone.0008930.s002.tif]

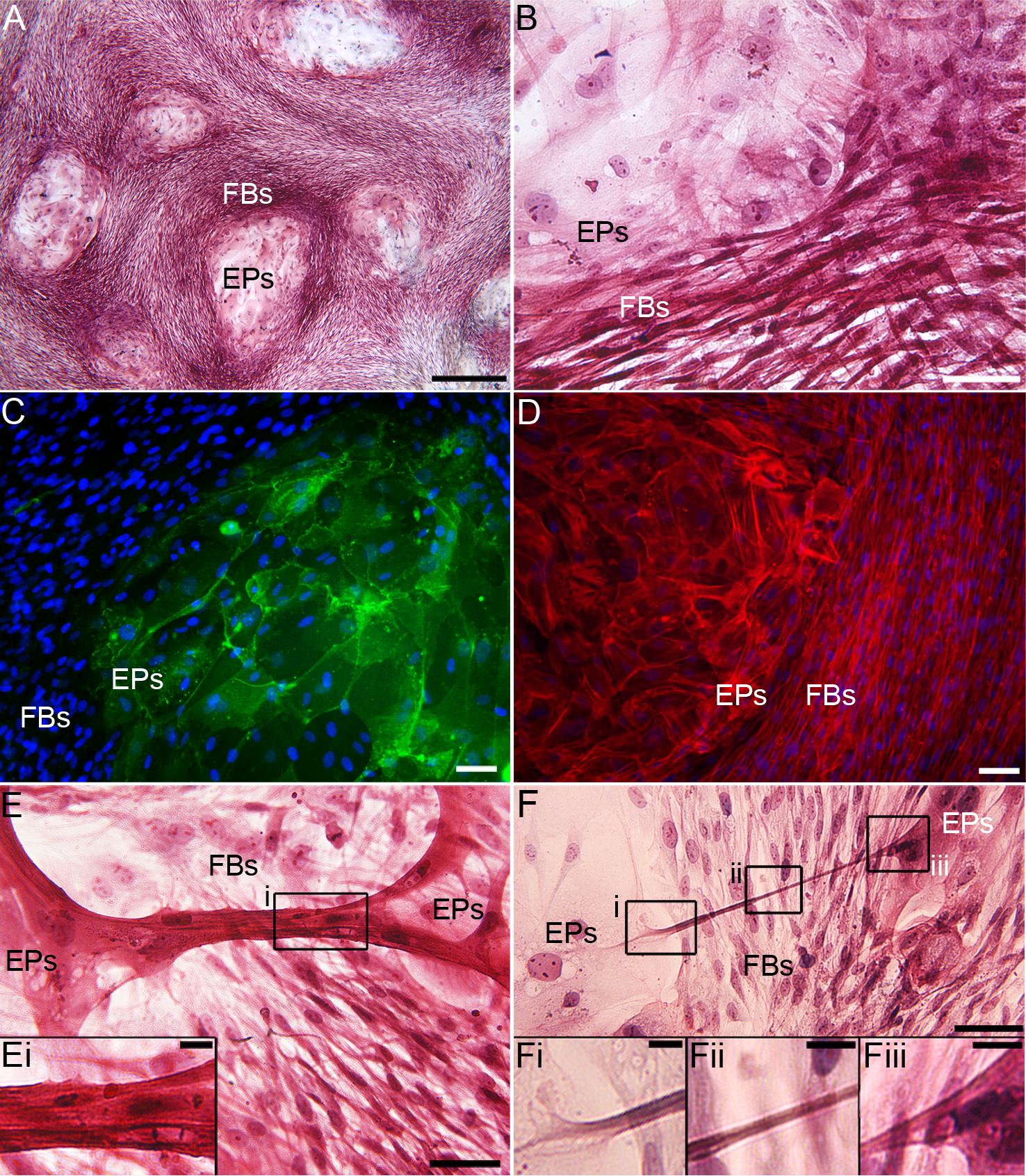

Supplement: Figure S3 — Morphology of EP bridges and EP islands in co-cultures of EPs and FBs. A–B: Trichrome staining in EPs/FBs of EP islands surrounded by FBs. C: Nucleus (blue) and E-cadherin (green) immunostaining displayed segregation of EPs and FBs. D: Staining of F-actin (red) in EPs and FBs. E–F: Trichome staining of EP bridges between EP islands with (E-Ei) and without (F-Fiii) nuclei. Scale bars: (A), 500 µm; (B–F), 50 µm; (Ei, Fi-Fiii), 10 µm. (6.16 MB TIF) [file pone.0008930.s003.tif]

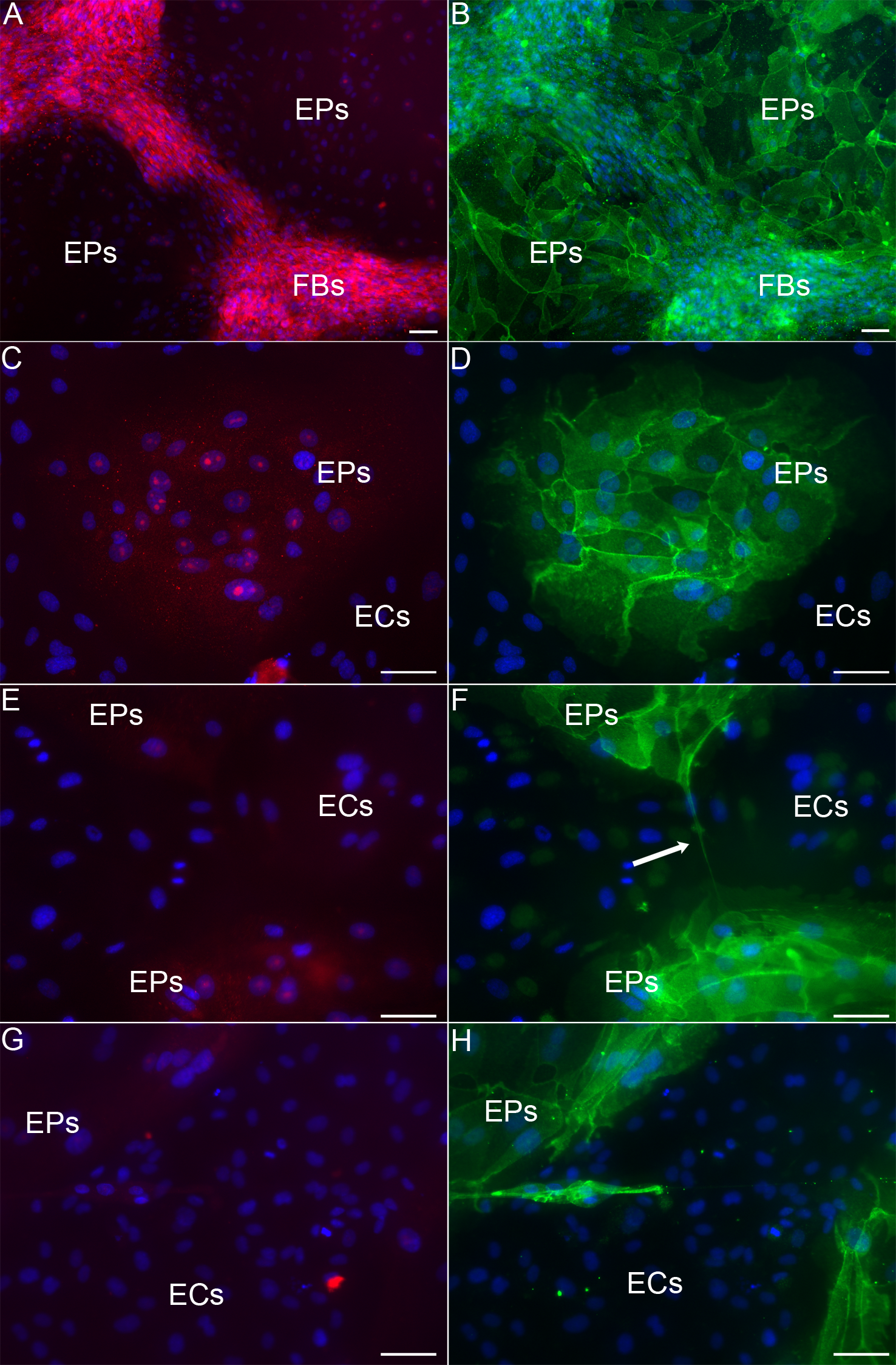

Supplement: Figure S4 — EP islands and EP bridges do not express the EMT marker α-smooth muscle actin. A–B: Nucleus (blue), α-smooth muscle actin (red, A), and E-cadherin (green, B) immunostaining showed only FBs expressed α-smooth muscle actin in EPs/FBs. C–H: EP islands (C–D) and EP bridges without (E–F) or with nuclei (G–H) did not express α-smooth muscle actin in EPs/ECs (arrow indicates EP bridge in panel F). Panels A, C, E, and G correspond to panels B, D, F, and H respectively. Scale bars: 50 µm. (4.28 MB TIF) [file pone.0008930.s004.tif]

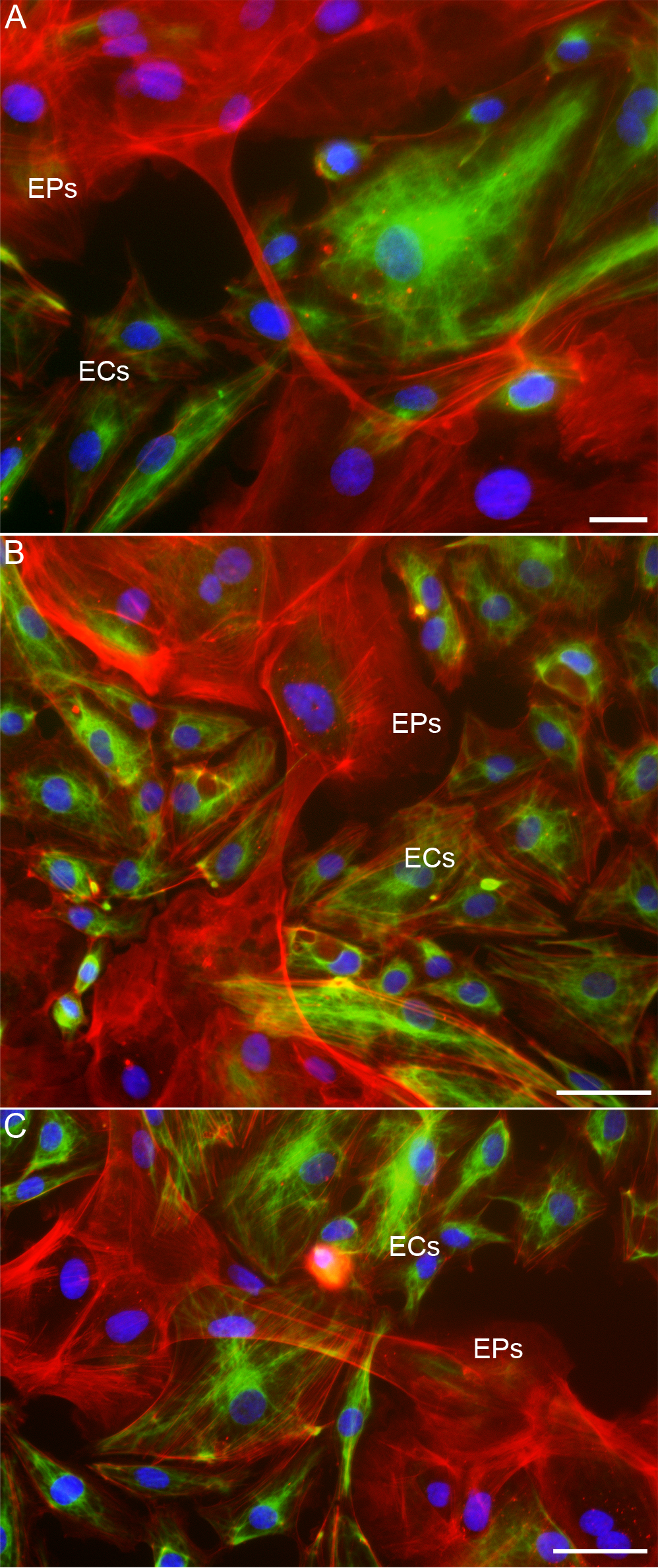

Supplement: Figure S5 — EP islands and EP bridges do not express the EMT marker vimentin. A–C: F-actin (red), vimentin (green), and nucleus (blue) immunostaining show EP bridges without (A–B) and with nuclei (C) do not express vimentin. Scale bars: 50 µm. (4.18 MB TIF) [file pone.0008930.s005.tif]

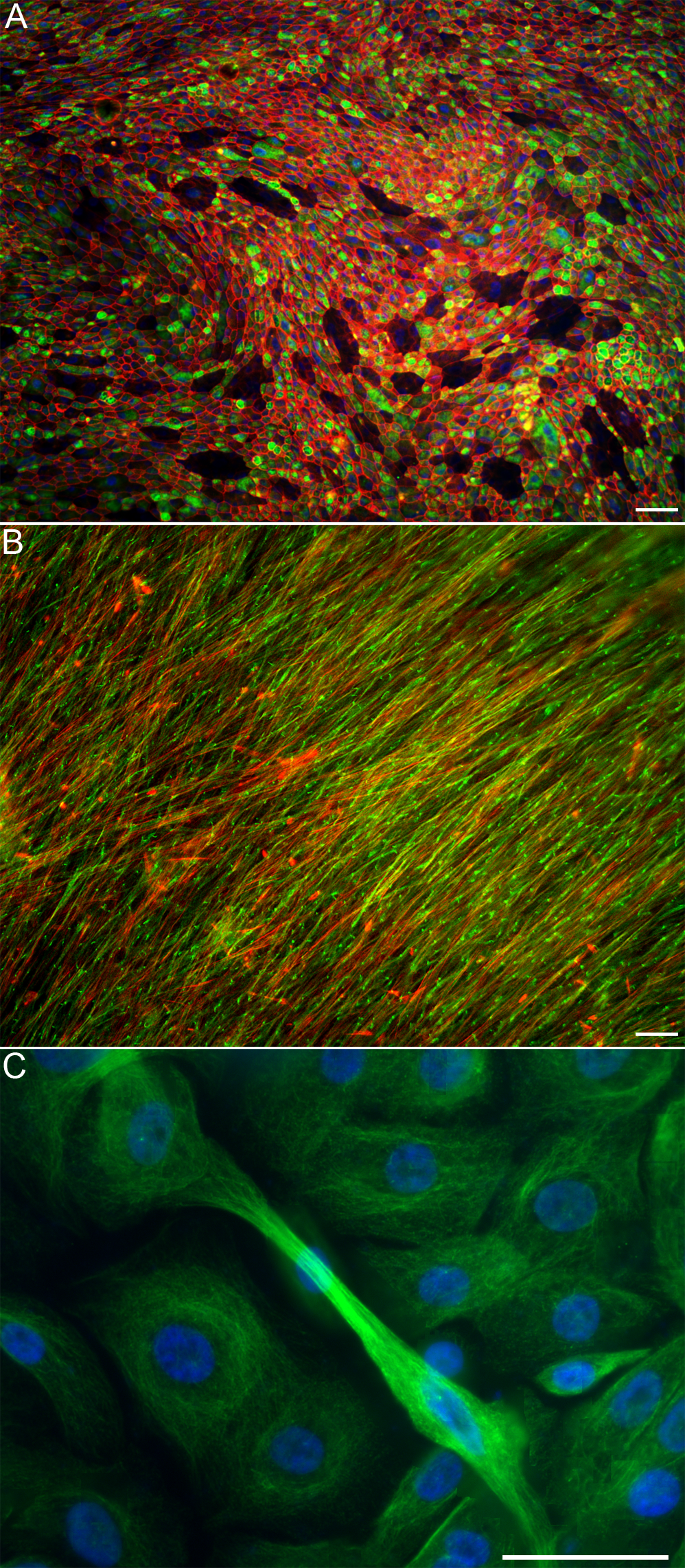

Supplement: Figure S6 — EP bridges form in air-liquid interfaces. A–C: EPs were grown on the apical surface of collagen IV-coated membranes and exposed to air, while FBs were grown on the basolateral side of the same membrane and grown in 1∶1 mixture of BEGM:DMEM (low glucose) with 5% FBS for 3 weeks. EPs (A) on the apical surface and FBs (B) basolateral surface of the same membrane were immunostained for microtubules (green), F-actin (red), and nuclei (blue), while (C) EP bridge formation was observed even with EPs exposed to air. Scale bars: 50 µm. (4.93 MB TIF) [file pone.0008930.s006.tif]

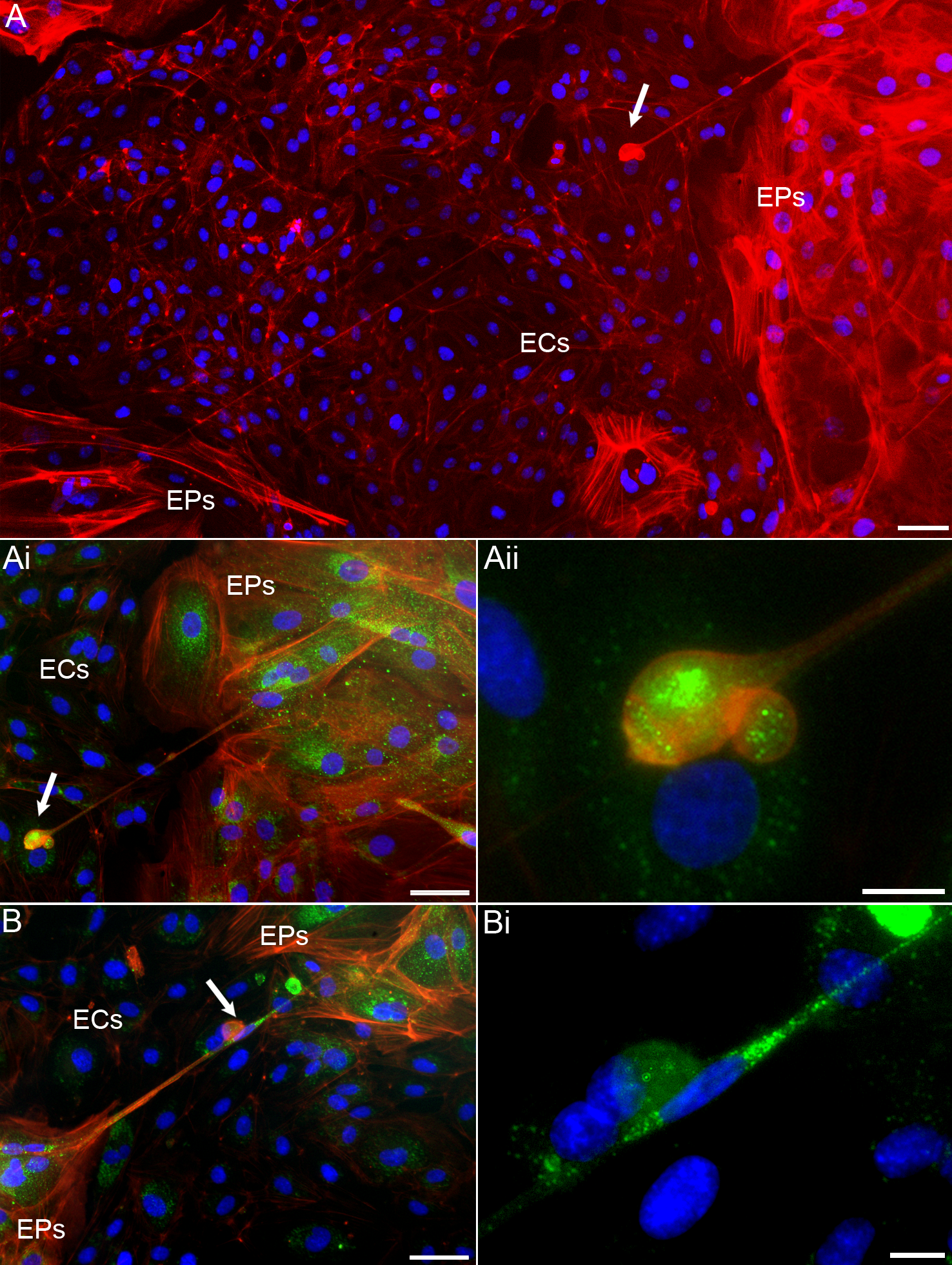

Supplement: Figure S7 — Golgi apparatuses localize to vesicle-like structures and cells in EP bridges. A: F-actin (red), Golgi marker (green), and nucleus (blue) immunostaining show a vesicle-like structure positive for Golgi in an 801 µm EP bridge in EPs/ECs. No nuclei were present in the EP bridge (A-Ai) or vesicle-like structure (Aii) (arrows indicate vesicle-like structure position). B: F-actin (red), Golgi marker (green), and nucleus (blue) immunostaining show a cell within an EP bridge (arrows indicate cell position). Scale bars: (A-Ai, B), 50 µm; (Aii, Bi), 10 µm. (4.15 MB TIF) [file pone.0008930.s007.tif]

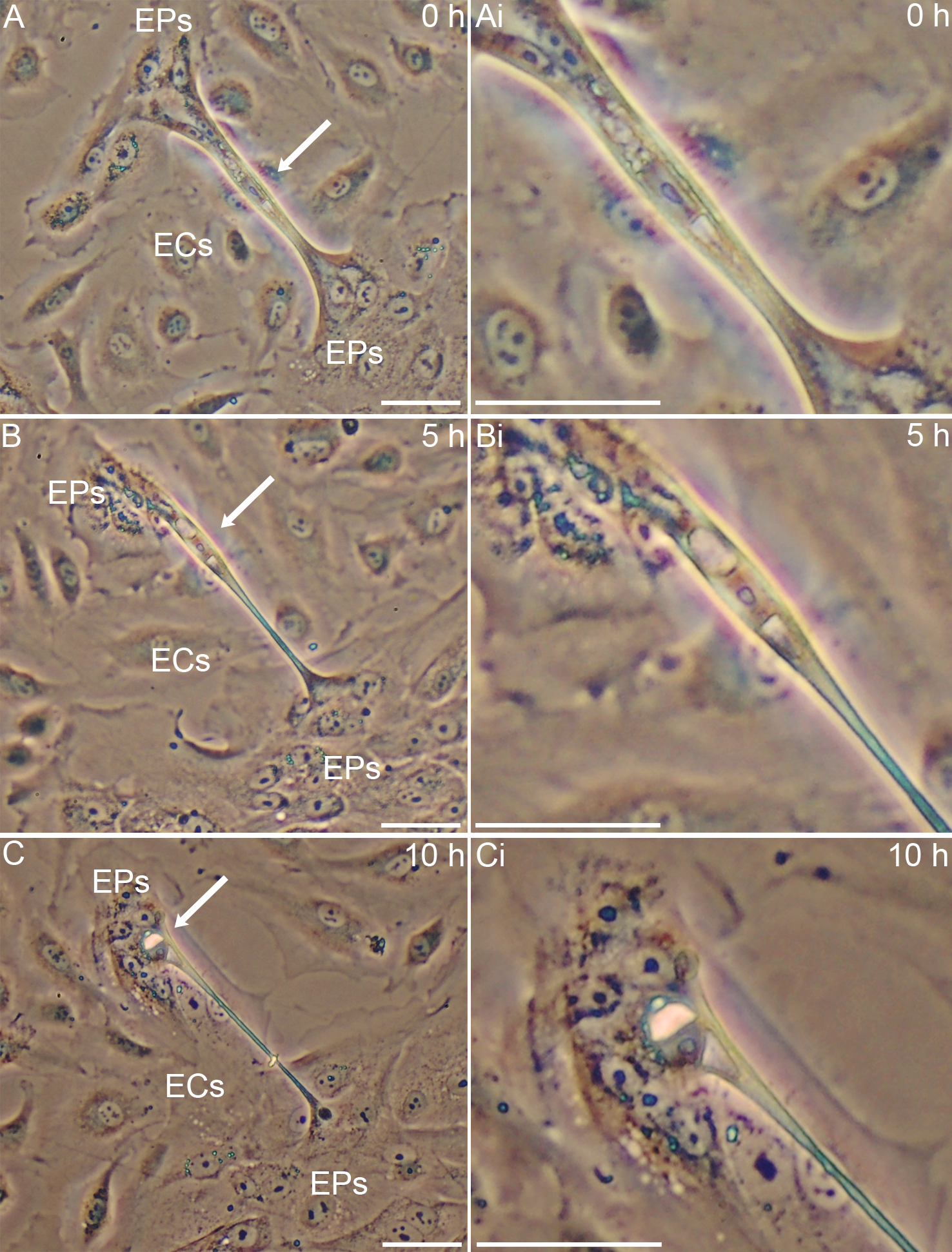

Supplement: Figure S8 — Cell migration through a type II EP bridge in EPs/ECs. A–C: Sequential images taken from Movie S2 show a cell in the middle of an EP bridge migrating towards an EP island in the top left of each image over 10 h. Panels Ai–Ci are magnified sections of A–C. Arrows indicate location of the cell within the EP bridge. Scale bars: 50 µm. (4.42 MB TIF) [file pone.0008930.s008.tif]

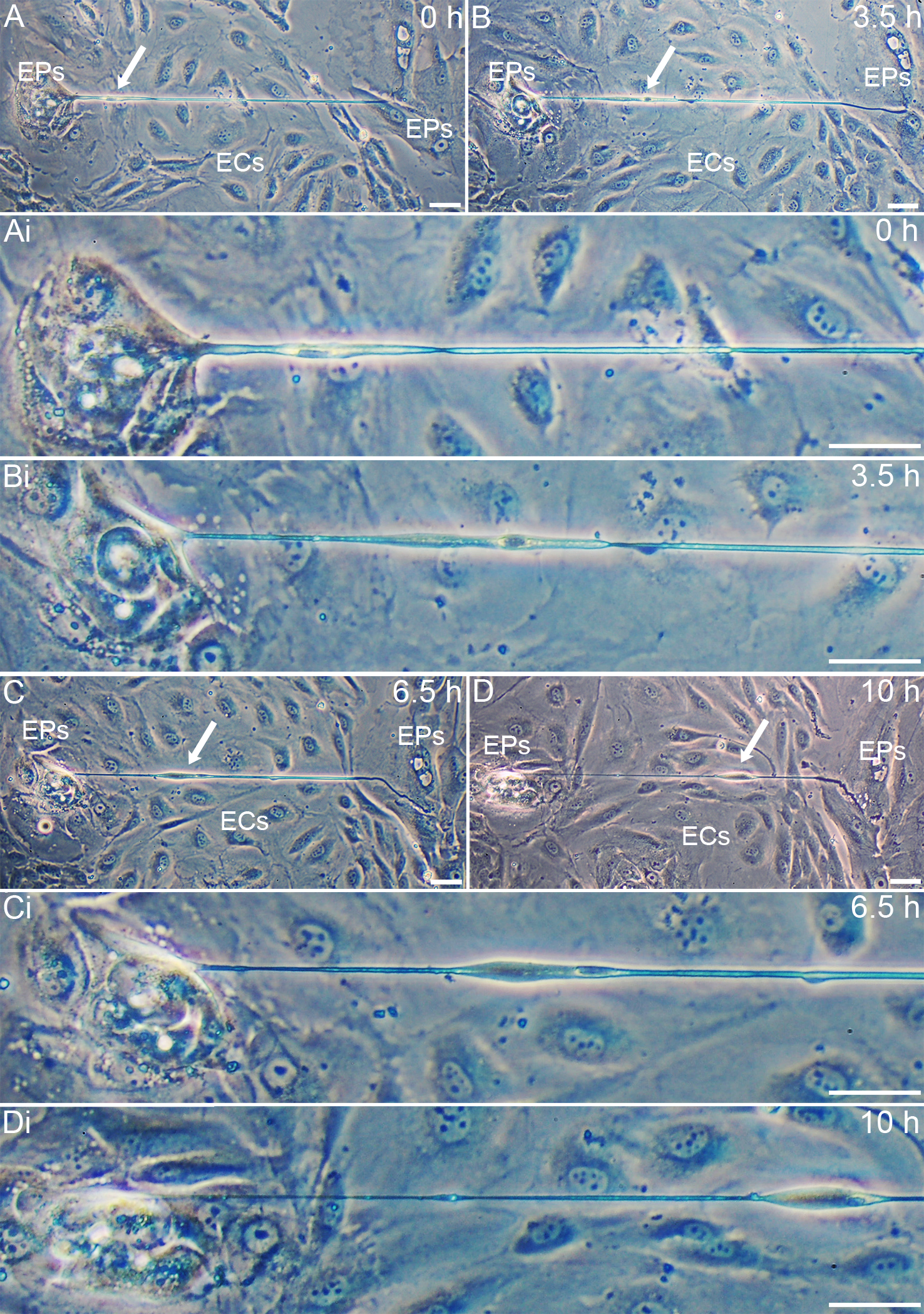

Supplement: Figure S9 — Cell migration through a type II EP bridge in EPs/ECs. A–D: Sequential images taken from Movie S3 show a cell within an EP bridge connected to an EP island on the left side migrating left to right toward the connected EP island on right side over 10 h. Panels Ai–Di are magnified sections of A–D. Arrows indicate location of the cell within the EP bridge. Scale bars: 50 µm. (6.37 MB TIF) [file pone.0008930.s009.tif]

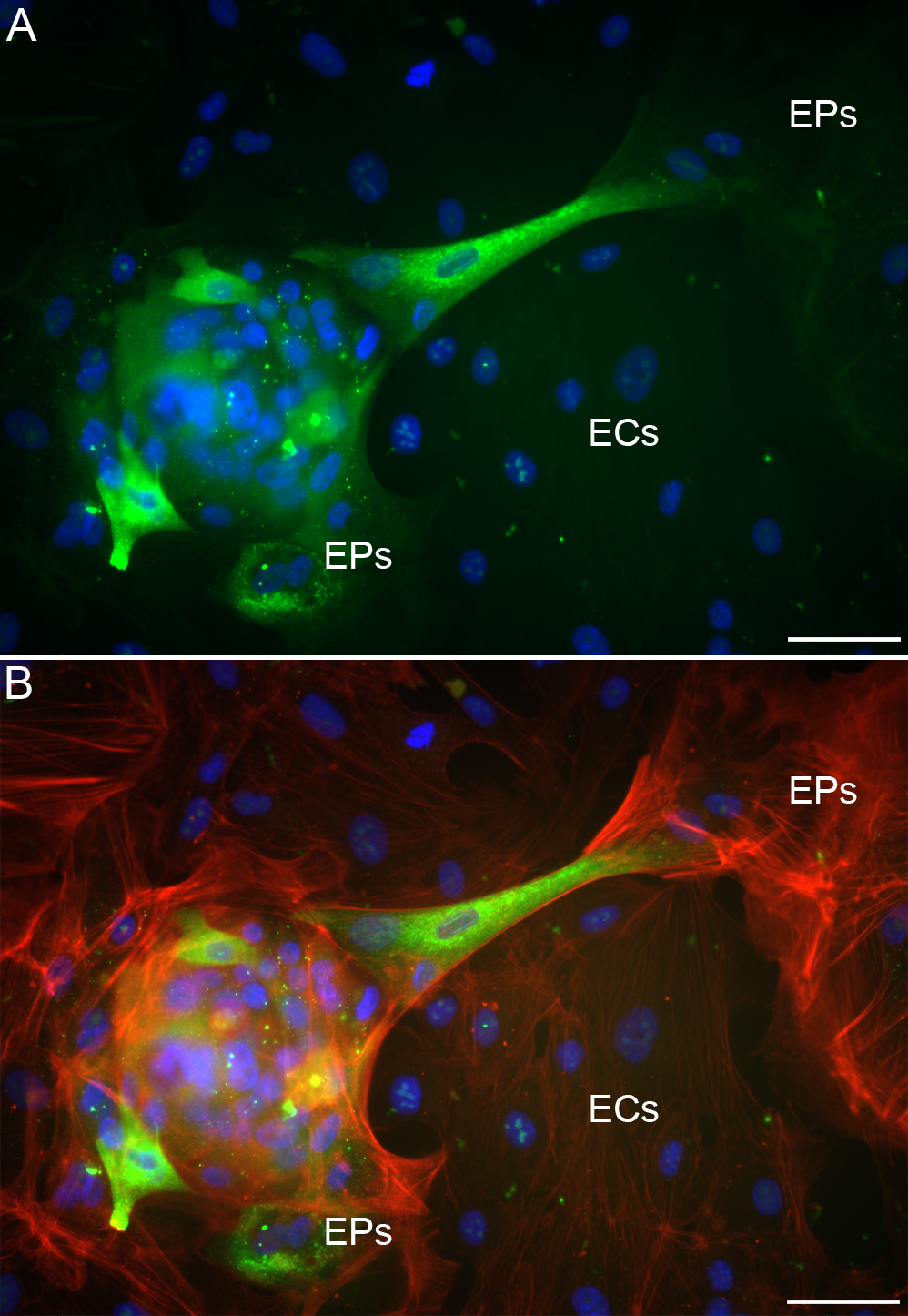

Supplement: Figure S10 — Clara cell within a type II EP bridge. A: Immunostaining for CC10 (green) and nuclei (blue) shows a Clara cell within an EP bridge in EPs/ECs. B: Addition of F-actin (red) to color composite in image A shows EP bridge and EP island architecture. Scale bars: 50 µm. (1.96 MB TIF) [file pone.0008930.s010.tif]

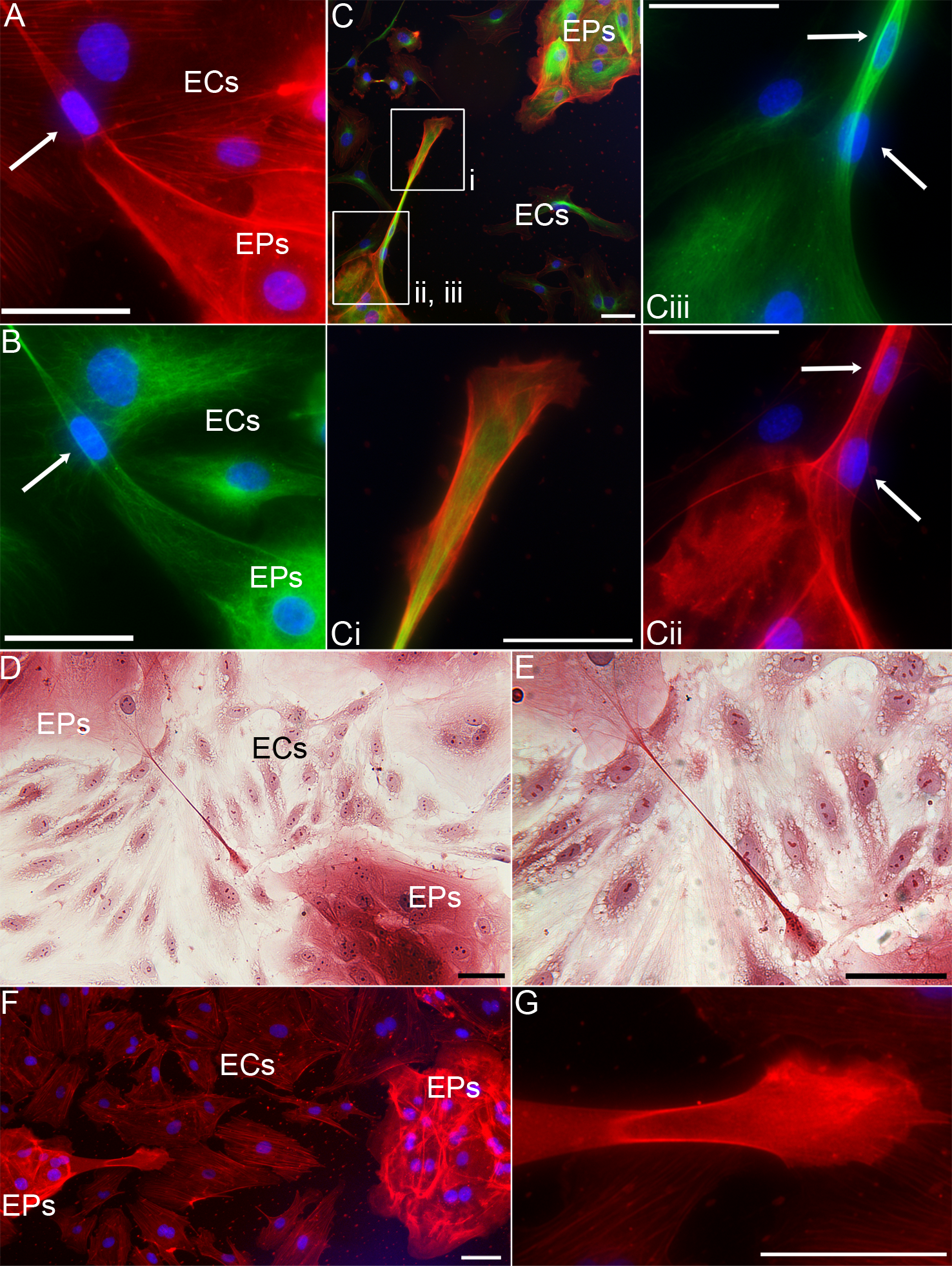

Supplement: Figure S11 — Architecture of EP filopodia-like extensions. A–B: Magnified images of Figure 4E (A) and 4Eii (B) where F-actin (red), nucleus (blue), and microtubule (green) immunostaining displayed a nucleus extending into the F-actin (A) and microtubule (B) structure of the filopodia-like extension in EPs/ECs (arrows indicate nucleus). C: Color composite of F-actin (red) and microtubule (green) expression of an EP bridge precursor from one EP island extending toward another EP island at the leading edge (Ci). F-actin (Cii) and microtubule (Ciii) expression at the base of the EP extension (arrows indicate nuclei). D–E: Trichome staining in EPs/ECs showed an EP filopodia-like structure extending from one EP island to another EP island (panels E is a magnified image of panel D). F–G: Wide EP filopodia-like structure extending out from one EP island toward another EP island (panel G is a magnified image of panel F). Scale bars: 50 µm. (4.85 MB TIF) [file pone.0008930.s011.tif]

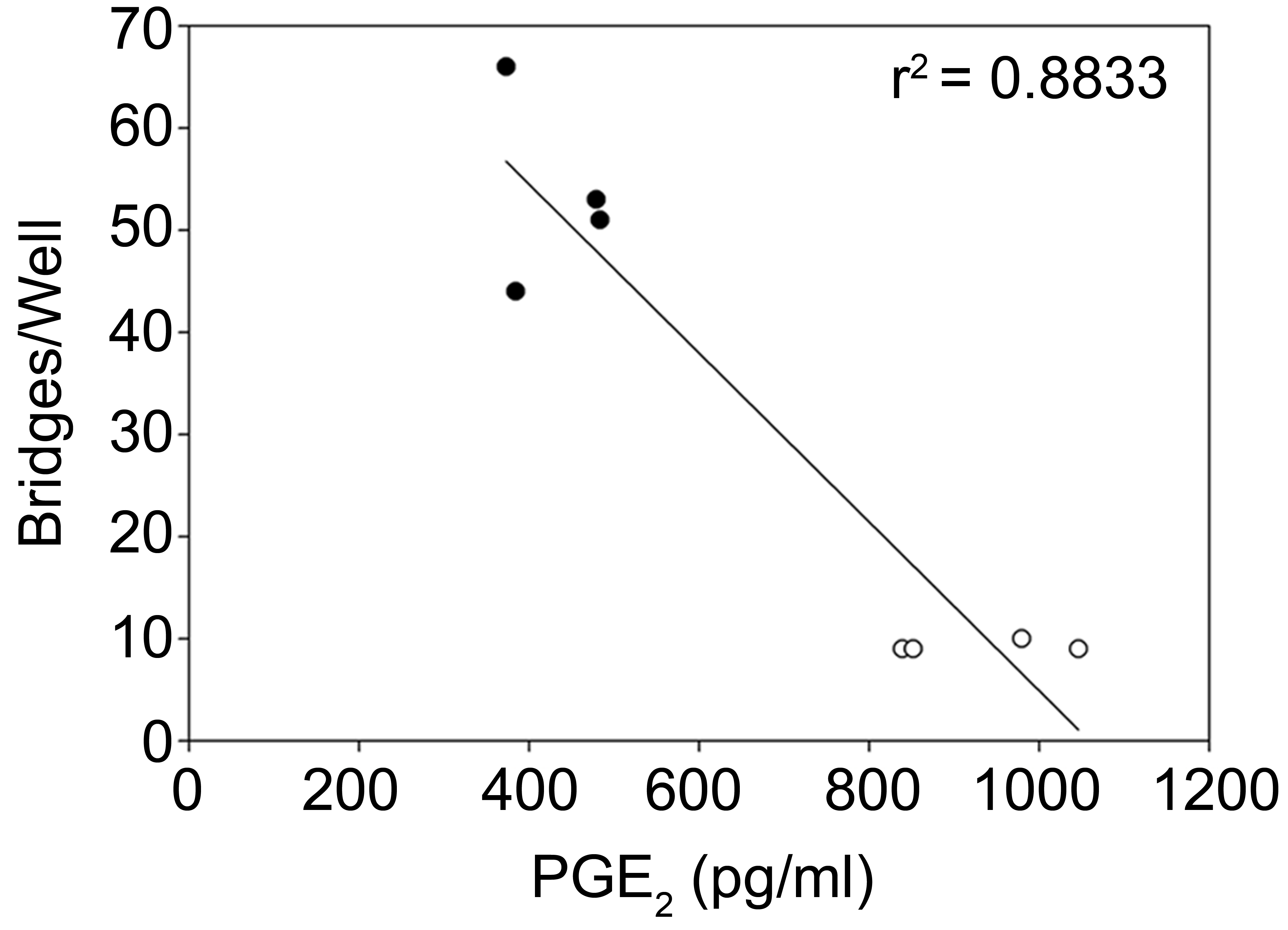

Supplement: Figure S12 — PGE2 expression affects EP bridges formation. EP bridge formation inversely correlates with PGE2 levels from 24 h conditioned media in co-cultures. Closed circles are EPs/FBs, open circles are EPs/ECs. (0.47 MB TIF) [file pone.0008930.s012.tif]
